# Harmonization of resting-state functional MRI data across multiple imaging sites via the separation of site differences into sampling bias and measurement bias

**DOI:** 10.1371/journal.pbio.3000042

**Published:** 2019-04-18

**Authors:** Ayumu Yamashita, Noriaki Yahata, Takashi Itahashi, Giuseppe Lisi, Takashi Yamada, Naho Ichikawa, Masahiro Takamura, Yujiro Yoshihara, Akira Kunimatsu, Naohiro Okada, Hirotaka Yamagata, Koji Matsuo, Ryuichiro Hashimoto, Go Okada, Yuki Sakai, Jun Morimoto, Jin Narumoto, Yasuhiro Shimada, Kiyoto Kasai, Nobumasa Kato, Hidehiko Takahashi, Yasumasa Okamoto, Saori C. Tanaka, Mitsuo Kawato, Okito Yamashita, Hiroshi Imamizu

**Affiliations:** 1 Brain Information Communication Research Laboratory Group, Advanced Telecommunications Research Institutes International, Kyoto, Japan; 2 Department of Neuropsychiatry, Graduate School of Medicine, The University of Tokyo, Tokyo, Japan; 3 Department of Molecular Imaging and Theranostics, National Institute of Radiological Sciences, National Institutes for Quantum and Radiological Science and Technology, Chiba, Japan; 4 Medical Institute of Developmental Disabilities Research, Showa University, Tokyo, Japan; 5 Department of Psychiatry and Neurosciences, Hiroshima University, Hiroshima, Japan; 6 Department of Psychiatry, Kyoto University Graduate School of Medicine, Kyoto, Japan; 7 Department of Radiology, IMSUT Hospital, Institute of Medical Science, The University of Tokyo, Tokyo, Japan; 8 Department of Radiology, Graduate School of Medicine, The University of Tokyo, Tokyo, Japan; 9 The International Research Center for Neurointelligence (WPI-IRCN) at the University of Tokyo Institutes for Advanced Study (UTIAS), Tokyo, Japan; 10 Division of Neuropsychiatry, Department of Neuroscience, Yamaguchi University Graduate School of Medicine, Yamaguchi, Japan; 11 Department of Psychiatry, Faculty of Medicine, Saitama Medical University, Saitama, Japan; 12 Department of Language Sciences, Tokyo Metropolitan University, Tokyo, Japan; 13 Department of Psychiatry, Graduate School of Medical Science, Kyoto Prefectural University of Medicine, Kyoto, Japan; 14 Brain Activity Imaging Center, ATR-Promotions Inc., Kyoto, Japan; 15 Center for Advanced Intelligence Project, RIKEN, Tokyo, Japan; 16 Department of Psychology, Graduate School of Humanities and Sociology, The University of Tokyo, Tokyo, Japan; University of Edinburgh, UNITED KINGDOM

## Abstract

When collecting large amounts of neuroimaging data associated with psychiatric disorders, images must be acquired from multiple sites because of the limited capacity of a single site. However, site differences represent a barrier when acquiring multisite neuroimaging data. We utilized a traveling-subject dataset in conjunction with a multisite, multidisorder dataset to demonstrate that site differences are composed of biological sampling bias and engineering measurement bias. The effects on resting-state functional MRI connectivity based on pairwise correlations because of both bias types were greater than or equal to psychiatric disorder differences. Furthermore, our findings indicated that each site can sample only from a subpopulation of participants. This result suggests that it is essential to collect large amounts of neuroimaging data from as many sites as possible to appropriately estimate the distribution of the grand population. Finally, we developed a novel harmonization method that removed only the measurement bias by using a traveling-subject dataset and achieved the reduction of the measurement bias by 29% and improvement of the signal-to-noise ratios by 40%. Our results provide fundamental knowledge regarding site effects, which is important for future research using multisite, multidisorder resting-state functional MRI data.

## Abstract 2

Acquiring and sharing large amounts of resting-state fMRI data from multiple imaging sites has recently become critical for bridging the gap between basic neuroscience research and clinical applications. A harmonization method using traveling-subject data achieved a reduction of between-site differences from a multisite dataset.

## Author summary

Recently, the importance of acquiring and sharing large amounts of resting-state functional magnetic resonance imaging (rs-fMRI) data from multiple geographical locations or sites has increased. However, differences in the data acquired from multiple sites create heterogeneities that present a barrier to the analysis. To properly manage these heterogeneous multisite data, it is important to have a deeper understanding of the origin of these between-site differences and to harmonize rs-fMRI data among sites. In this study, we demonstrate that site differences are composed of biological sampling bias (differences between the participant groups) and engineering measurement bias (differences in the properties of the MRI scanners used). We found that the effects of both types of bias on rs-fMRI functional connectivity were greater than or equal to those driven by psychiatric disorders. Furthermore, our results identified the specific properties of MRI scanners that affect the rs-fMRI connectivity. To overcome the limitations associated with site differences, we used a traveling-subject dataset, wherein multiple participants travel to multiple sites to assess measurement bias by controlling for participant effects between sites. Our results indicated that the traveling-subject dataset can help the proper harmonization of rs-fMRI data between sites.

## Introduction

Acquiring and sharing large amounts of neuroimaging data have recently become critical for bridging the gap between basic neuroscience research and clinical applications, such as the diagnosis and treatment of psychiatric disorders (Human Connectome Project [HCP] [1] [http://www.humanconnectomeproject.org/]; Human Brain Project [https://www.humanbrainproject.eu/en/]; UK Biobank [http://www.ukbiobank.ac.uk/]; and Strategic Research Program for Brain Sciences [SRPBS] [2] [https://bicr.atr.jp/decnefpro/]) [3–5]. When collecting large amounts of data associated with psychiatric disorders, it is necessary to acquire images from multiple sites because it is nearly impossible for a single site to collect a large amount of neuroimaging data from many participants (Connectomes Related to Human Disease [CRHD] [https://www.humanconnectome.org/disease-studies], Autism Brain Imaging Data Exchange [ABIDE], and SRPBS) [2, 6–8]. In 2013, the Japan Agency for Medical Research and Development (AMED) organized the Decoded Neurofeedback (DecNef) Project. The project determined a unified imaging protocol on 28 February 2014 (https://bicr.atr.jp/rs-fmri-protocol-2/) and has collected multisite resting-state functional magnetic resonance imaging (rs-fMRI) data using 14 scanners across eight research institutes for the last 5 y. The collected dataset encompasses 2,239 participants and five disorders and is publicly shared through the SRPBS multisite multidisorder database (https://bicr-resource.atr.jp/decnefpro/). This project has enabled the identification of resting-state functional connectivity MRI (rs-fcMRI)-based biomarkers of several psychiatric disorders that can be generalized to completely independent cohorts [2, 8–10]. However, a multisite dataset with multiple disorders raises difficult problems that are not present in a single site–based dataset (e.g., HCP and UK Biobank). That is, our experience in the SRPBS database demonstrated difficulties in differences due to scanner type, imaging protocol, and patient demographics [10–13], even when a unified protocol was determined. Moreover, unpredictable differences in participant population can often exist between sites. Therefore, researchers must work with heterogeneous neuroimaging data. In particular, site differences represent a barrier when extracting disease factors by applying machine-learning techniques to such heterogeneous data [14] because disease factors tend to be confounded with site factors [2, 8, 10–13, 15]. This confounding occurs because a single site (or hospital) is apt to sample only a few types of psychiatric disorders (e.g., primarily schizophrenia [SCZ] and autism spectrum disorder [ASD] from sites A and B, respectively). Although robust generalization across sites could be possible as long as the pattern of the disease factors is sufficiently different from the pattern due to the site differences [11], these factors depend on dataset and type of disease. To properly manage these heterogeneous data, it is important for them to be harmonized between sites [16–19]. Moreover, a deeper understanding of these site differences is essential for efficient harmonization of the data.

Site differences consist of two types of biases: engineering bias (measurement bias) and biological bias (sampling bias). Measurement bias includes differences in the properties of MRI scanners—such as imaging variables, field strength, MRI manufacturers, and scanner models—whereas sampling bias refers to differences in participant groups between sites. In this study, we used the word “bias” to indicate a systematic shift from the global population at a given site or with given imaging variables. Previous studies have investigated the effect of measurement bias on resting-state functional connectivity by using a traveling-subject design [20], wherein multiple participants travel to multiple sites to assess measurement bias [7]. By contrast, researchers to date have only speculated about sampling bias. For example, differences in the clinical characteristics of patients examined at different sites are presumed to underlie the stagnant accuracy of certain biomarkers, even after combining data from multiple sites [12]. Furthermore, to the best of our knowledge, no study has mathematically defined sampling bias or conducted quantitative analyses of its effect size, which is likely because the decomposition of site differences into measurement bias and sampling bias is a complex process. To achieve this aim, we combined a separate traveling-subject rs-fMRI dataset with the SRPBS multidisorder dataset. Simultaneous analysis of the datasets enabled us to divide site differences into measurement bias and sampling bias and quantitatively compare their effect sizes on resting-state functional connectivity with those of psychiatric disorders.

Furthermore, our detailed analysis of measurement and sampling biases enabled us to investigate the origin of each bias in multisite datasets for the first time. For measurement bias, we quantitatively compared the magnitude of the effects between different imaging variables, fMRI manufacturers, and the number of channels per coil in each fMRI scanner. We further examined two alternative hypotheses regarding the mechanisms underlying sampling bias: one hypothesis assumes that each site samples subjects from a common population. In this situation, sampling bias occurs because of the random sampling of subjects, which results in incidental differences in the patients’ characteristics among the sites. The second hypothesis assumes that each site samples subjects from different subpopulations. In this situation, sampling bias occurs because of sampling from subpopulations with different characteristics. For example, assume multiple sites plan to collect data from the same population of patients with major depressive disorder (MDD). Subtypes of MDD exist within the population, such as atypical depression and melancholic depression [21, 22]; therefore, one subpopulation may contain a large proportion of patients with atypical depression, whereas another subpopulation may contain a large proportion of patients with melancholic depression. Therefore, in some instances, atypical depression may be more frequent among patients at site A, whereas melancholic depression may be more frequent among patients at site B. The basic protocol for collecting large-scale datasets differs between these two hypotheses; thus, it is necessary to determine the hypothesis that most appropriately reflects the characteristics of the SRPBS dataset. In the former situation, one would simply need to collect data from a large number of individuals, even with a small number of sites. In the latter situation, a larger number of sites would be required to obtain truly representative data.

To overcome the limitations associated with site differences, we developed a novel harmonization method that enabled us to subtract only the measurement bias by using a traveling-subject dataset. We investigated the extent that our proposed method could reduce measurement bias and improve the signal-to-noise ratio. We compared its performance to those of other commonly used harmonization methods. All data utilized in this study can be downloaded publicly from the DecNef Project Brain Data Repository at https://bicr-resource.atr.jp/decnefpro/.

## Results

### Datasets

We used two rs-fMRI datasets: (1) the SRPBS multidisorder dataset and (2) a traveling-subject dataset.

#### SRPBS multidisorder dataset

**Fig 1:**
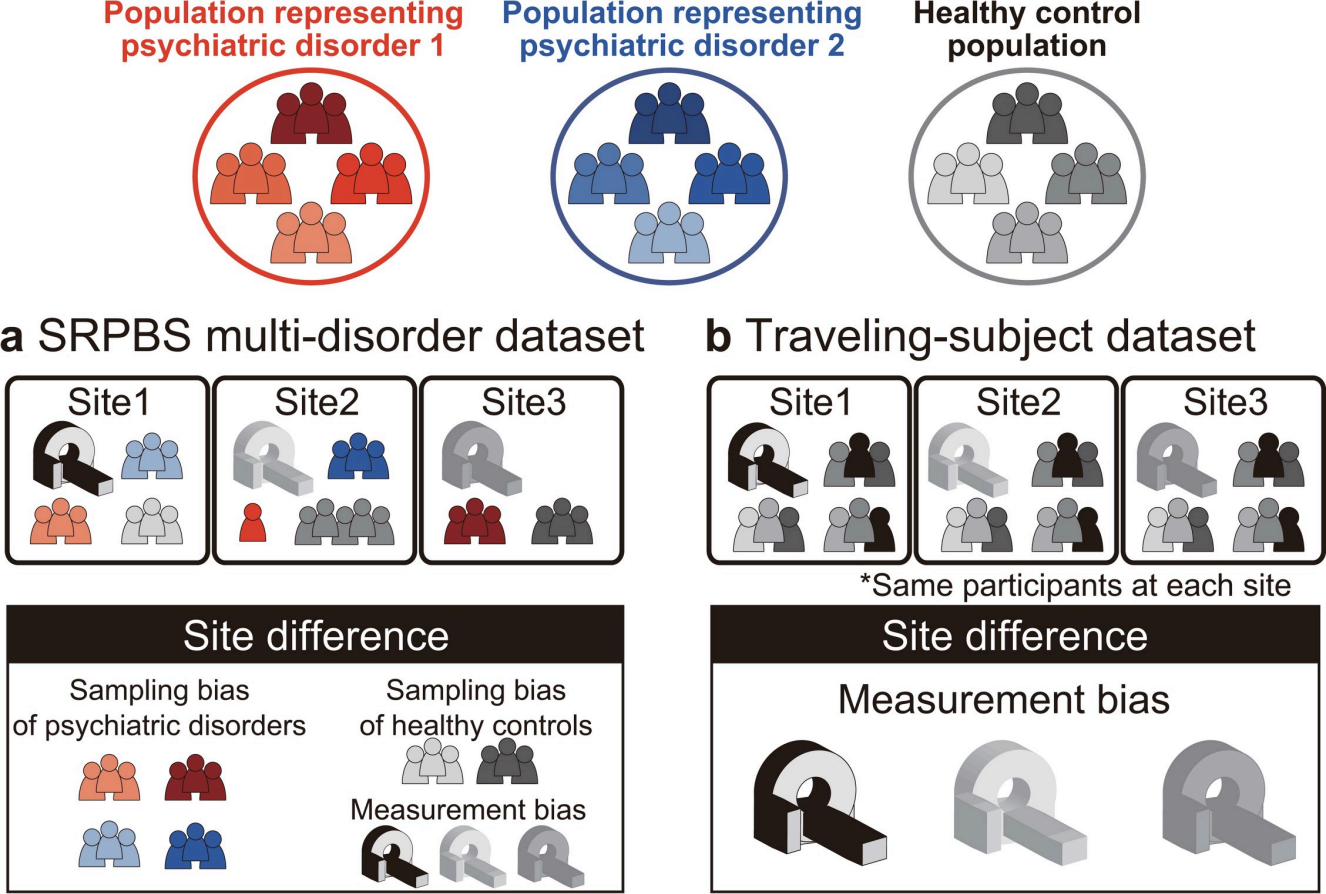
Schematic examples illustrating the two main datasets. (a) The SRPBS multidisorder dataset includes patients with psychiatric disorders and healthy controls. The number of patients and scanner types differed among sites. Thus, site differences consist of sampling bias and measurement bias. (b) The traveling-subject dataset includes only healthy controls, and the participants were the same across all sites. Thus, site differences consist of measurement bias only. SRPBS, Strategic Research Program for Brain Sciences.

This dataset included patients with five different disorders and healthy controls (HCs) who were examined at nine sites belonging to eight research institutions. A total of 805 participants were included: 482 HCs from nine sites, 161 patients with MDD from five sites, 49 patients with ASD from one site, 65 patients with obsessive-compulsive disorder (OCD) from one site, and 48 patients with SCZ from three sites (Table 1). The rs-fMRI data were acquired using a unified imaging protocol at all but three sites (Table 2; https://bicr.atr.jp/rs-fmri-protocol-2/). Site differences in this dataset included both measurement and sampling biases (Fig 1A). For bias estimation, we only used data obtained using the unified protocol. (Patients with OCD were not scanned using this unified protocol; therefore, a disorder factor could not be estimated for OCD.)

**Table 1:** Demographic characteristics of patients included in the SRPBS multidisorder dataset.

**Site**	**HC**			**MDD**			**ASD**			**OCD**			**SCZ**			**ALL**			*1
	Number	Male /Female	Age (y)	Number	Male /Female	Age (y)	Number	Male /Female	Age (y)	Number	Male /Female	Age (y)	Number	Male /Female	Age (y)	Number	Male /Female	Age (y)	
ATT	31	28/3	23.0 ± 1.9	0	-	-	0	-	-	0	-	-	0	-	-	31	28/3	23.0 ± 1.9	✔
ATV	77	60/17	22.6 ± 2.0	0	-	-	0	-	-	0	-	-	0	-	-	77	60/17	22.6 ± 2.0	✔
HUH	66	29/37	34.6 ± 13.0	57	25/32	43.3 ± 12.2	0	-	-	0	-	-	0	-	-	123	54/69	38.6 ± 13.3	-
HKH	29	12/17	45.4 ± 9.5	23	10/13	43.6 ± 11.6	0	-	-	0	-	-	0	-	-	52	22/30	44.6 ± 10.5	-
COI	10	5/5	43.5 ± 13.5	38	18/20	44.0 ± 11.0	0	-	-	0	-	-	0	-	-	48	23/25	43.9 ± 11.4	✔
KPM	52	28/24	29.1 ± 7.3	0	-	-	0	-	-	65	30/35	31.9 ± 9.8	0	-	-	117	58/59	30.6 ± 8.8	-
KUT	35	18/17	36.3 ± 8.9	9	5/4	45.2 ± 15.9	0	-	-	0	-	-	22	11/11	40.4 ± 8.4	66	34/32	38.9 ± 10.2	✔
SWA	40	32/8	30.9 ± 8.5	0	-	-	49	45/4	32.9 ± 8.1	0	-	-	12	11/1	41.8 ± 9.2	101	88/13	33.2 ± 8.9	✔
UTO	142	72/70	29.7 ± 11.0	34	16/18	38.5 ± 9.9	0	-	-	0	-	-	14	7/7	33.3 ± 14.0	190	95/95	31.6 ± 11.5	✔
Summary	482	284/198	30.6 ± 10.9	161	74/87	42.6 ± 11.7	49	45/4	32.9 ± 8.1	65	30/35	31.9 ± 9.8	48	29/19	38.7 ± 10.8	805	462/343	33.7 ± 11.9	-

*1: Participants scanned using the unified protocol.

Abbreviations: ASD, autism spectrum disorder; ATT, Siemens TimTrio scanner at Advanced Telecommunications Research Institute International; ATV, Siemens Verio scanner at Advanced Telecommunications Research Institute International; COI, Center of Innovation in Hiroshima University; HC, healthy control; HKH, Hiroshima Kajikawa Hospital; HUH, Hiroshima University Hospital; KPM, Kyoto Prefectural University of Medicine; KUT, Siemens TimTrio scanner at Kyoto University; MDD, major depressive disorder; OCD, obsessive-compulsive disorder; SCZ, schizophrenia; SRPBS, Strategic Research Program for Brain Sciences; SWA, Showa University; UTO, University of Tokyo.

**Table 2:** Imaging protocols for resting-state fMRI in the SRPBS multidisorder dataset.

Site	ATT	ATV	COI	HUH	HKH	KPM	SWA	KUT	UTO
MRI scanner	Siemens TimTrio	Siemens Verio	Siemens Verio	GE SignaHDxt	Siemens Spectra	Philips Achieva	Siemens Verio	Siemens TimTrio	GE MR750w
Magnetic field strength	3.0 T	3.0 T	3.0 T	3.0 T	3.0 T	3.0 T	3.0 T	3.0 T	3.0 T
Number of channels per coil	12	12	12	8	12	8	12	32	24
Field of view (mm)	212 × 212	212 × 212	212 × 212	256 × 256	192 × 192	192 × 192	212 × 212	212 × 212	212 × 212
Matrix	64 × 64	64 × 64	64 × 64	64 × 64	64 × 64	64 × 64	64 × 64	64 × 64	64 × 64
Number of slices	40 or 39	39	40	32	38	39	40	40	40
Number of volumes	240	240	240	143	107	194	240	240	240
In-plane resolution (mm)	3.3125 × 3.3125	3.3125 × 3.3125	3.3125 × 3.3125	4.0 × 4.0	3.0 × 3.0	3.0 × 3.0	3.3125 × 3.3125	3.3125 × 3.3125	3.3125 × 3.3125
Slice thickness (mm)	3.2	3.2	3.2	4.0	3.0	3.0	3.2	3.2	3.2
Slice gap (mm)	0.8	0.8	0.8	0	0	0	0.8	0.8	0.8
TR (ms)	2,500	2,500	2,500	2,000	2,700	2,000	2,500	2,500	2,500
TE (ms)	30	30	30	27	31	30	30	30	30
Total scan time (min:s)	10:00	10:00	10:00	5:00	5:00	6:30	10:00	10:00	10:00
Flip angle (deg)	80	80	80	90	90	80	80	80	80
Slice acquisition order	Ascending	Ascending	Ascending	Ascending (Interleaved)	Ascending	Ascending	Ascending	Ascending	Ascending
Phase encoding	PA	PA	AP	PA	AP	AP	PA	PA	PA
Eye closed/fixate	Fixate	Fixate	Fixate	Fixate	Fixate	Closed	Fixate	Fixate	Fixate

Abbreviations: AP, anterior to posterior; ATT, Siemens TimTrio scanner at Advanced Telecommunications Research Institute International; ATV, Siemens Verio scanner at Advanced Telecommunications Research Institute International; COI, Center of Innovation in Hiroshima University; fMRI, functional magnetic resonance imaging; HKH, Hiroshima Kajikawa Hospital; HUH, Hiroshima University Hospital; KPM, Kyoto Prefectural University of Medicine; KUT, Siemens TimTrio scanner at Kyoto University; PA, posterior to anterior; SRPBS: Strategic Research Program for Brain Sciences; SWA, Showa University; TR, repetition time; TE, echo time; UTO, University of Tokyo.

#### Traveling-subject dataset

We acquired a traveling-subject dataset to estimate measurement bias across sites in the SRPBS dataset. Nine healthy participants (all men; age range: 24–32 y; mean age: 27 ± 2.6 y) were scanned at each of 12 sites, which included the nine sites in the SRPBS dataset, and produced a total of 411 scan sessions (see “Participants” in the Methods section). Although we had attempted to acquire this dataset using the same imaging protocol as that in the SRPBS multidisorder dataset, there were some differences in the imaging protocol across sites because of limitations in variable settings or the scanning conventions of each site (S1 Table). There were two phase-encoding directions (posterior to anterior [P→A] and anterior to posterior [A→P]), three MRI manufacturers (Siemens, GE, and Philips), four numbers of channels per coil (8, 12, 24, and 32), and seven scanner types (TimTrio, Verio, Skyra, Spectra, MR750W, SignaHDxt, and Achieva). Site differences in this dataset included measurement bias only as the same nine participants were scanned across the 12 sites (Fig 1B).

### Calculation of rs-fMRI functional connectivity

We computed the region of interest (ROI)-based pairwise correlations as a measure of functional connectivity. For each participant, the temporal correlations of rs-fMRI blood-oxygen-level dependent (BOLD) signals between pairs of ROIs were computed after averaging each voxelwise BOLD signal in each ROI. There are some candidates for the measure of functional connectivity such as the tangent method and partial correlation [11, 23]; however, we used Pearson’s correlation coefficients because they have been the most commonly used values in previous studies. Functional connectivity was defined based on a functional brain atlas consisting of 268 nodes (regions) covering the whole brain, which has been widely utilized in previous studies [20, 24–26]. The Fisher’s *z*-transformed Pearson’s correlation coefficients between the preprocessed BOLD signal time courses of each possible pair of nodes were calculated and used to construct 268 × 268 symmetrical connectivity matrices in which each element represents a connection strength, or edge, between two nodes. We used 35,778 connectivity values [(268 × 267)/2] of the lower triangular part of the connectivity matrix. To briefly investigate any site effect on functional connectivity, we applied a one-way ANOVA with Site (9 sites) as a factor to the functional connections in the SRPBS multidisorder dataset and recorded the number of significant differences between sites. We set the threshold to *p* < 0.05, after Bonferroni correction. As a result, >30% of all connections (11,888/35,778) were significantly different between sites.

### Bias estimation

To quantitatively investigate the site differences in the rs-fcMRI data, we identified measurement biases, sampling biases, and disorder factors. We defined measurement bias for each site as a deviation of the connectivity value for each functional connection from its average across all sites. We assumed that the sampling biases of the HCs and patients with psychiatric disorders differed from one another. Therefore, we calculated the sampling biases for each site separately for HCs and patients with each disorder. Disorder factors were defined as deviations from the HC values. Sampling biases were estimated for patients with MDD and SCZ because only these patients were sampled at multiple sites. Disorder factors were estimated for MDD, SCZ, and ASD because patients with OCD were not scanned using the unified protocol.

It is difficult to separate site differences into measurement and sampling biases using the SRPBS multidisorder dataset alone because these two types of bias covaried across sites. Different samples (participants) were scanned using different variables (scanners and imaging protocols). In contrast, the traveling-subject dataset included only measurement bias because the participants were fixed. By combining the traveling-subject dataset with the SRPBS multidisorder dataset, we simultaneously estimated measurement bias and sampling bias as different factors are affected by different sites. We utilized a constrained linear regression model to assess the effects of both types of bias and disorder factors on functional connectivity, as follows. In the regression model for the SRPBS multidisorder dataset, the connectivity values of each participant in the SRPBS multidisorder dataset were composed of the sum of the average connectivity values across all participants and all sites at baseline, measurement bias, sampling bias, and disorder factors. The combined effect of participant factors (individual difference) and scan-to-scan variations was regarded as noise. In the regression model for the traveling-subject dataset, the connectivity values of each participant for a specific scan in the traveling-subject dataset were composed of the sum of the average connectivity values across all participants and all sites, participant factors, and measurement bias. Scan-to-scan variation was regarded as noise. For each participant, we defined the participant factor as a deviation of connectivity values from the average across all participants. We estimated all biases and factors by simultaneously fitting the aforementioned two regression models to the functional connectivity values of the two different datasets. For this regression analysis, we used data from participants scanned using a unified imaging protocol in the SRPBS multidisorder dataset and from all participants in the traveling-subject dataset. In summary, each bias or each factor was estimated as a vector that included a dimension reflecting the number of connectivity values (35,778). Vectors included in our further analyses are those for measurement bias at 12 sites, sampling bias of HCs at six sites, sampling bias for patients with MDD at three sites, sampling bias for patients with SCZ at three sites, participant factors of nine traveling subjects, and disorder factors for MDD, SCZ, and ASD. Note that since patients with ASD were scanned at one site, we could not estimate the sampling bias of ASD. Thus, the sampling bias was included in the disorder factor of ASD. For each connectivity, the regression model can be written as follows:

$$Connectivity={x_{m}}^{T}m+{x_{s_{hc}}}^{T}s_{hc}+{x_{s_{mdd}}}^{T}s_{mdd}+{x_{s_{scz}}}^{T}s_{scz}+{x_{d}}^{T}d+{x_{p}}^{T}p+const+e,$$

where ***m*** represents the measurement bias (12 sites × 1), ***s***_***hc***_ represents the sampling bias of HCs (6 sites × 1), ***s***_***mdd***_ represents the sampling bias of patients with MDD (3 sites × 1), ***s***_***scz***_ represents the sampling bias of patients with SCZ (3 sites × 1), ***d*** represents the disorder factor (3 × 1), ***p*** represents the participant factor (9 traveling subjects × 1), *const* represents the average functional connectivity value across all participants from all sites, and $e∼N(0,\gamma^{-1})$ represents noise. $x_{m},x_{s_{hc}},x_{s_{mdd}},x_{s_{scz}},x_{d},x_{p}$ are vectors represented by 1-of-K binary coding (more details are reported in “Estimation of biases and factors” in the Methods section). To eliminate the uncertainty of the constant term, we estimated measurement bias and each sampling bias by imposing constraints so that their average across sites would be 0.

### Quantification of site differences

**Fig 2:**
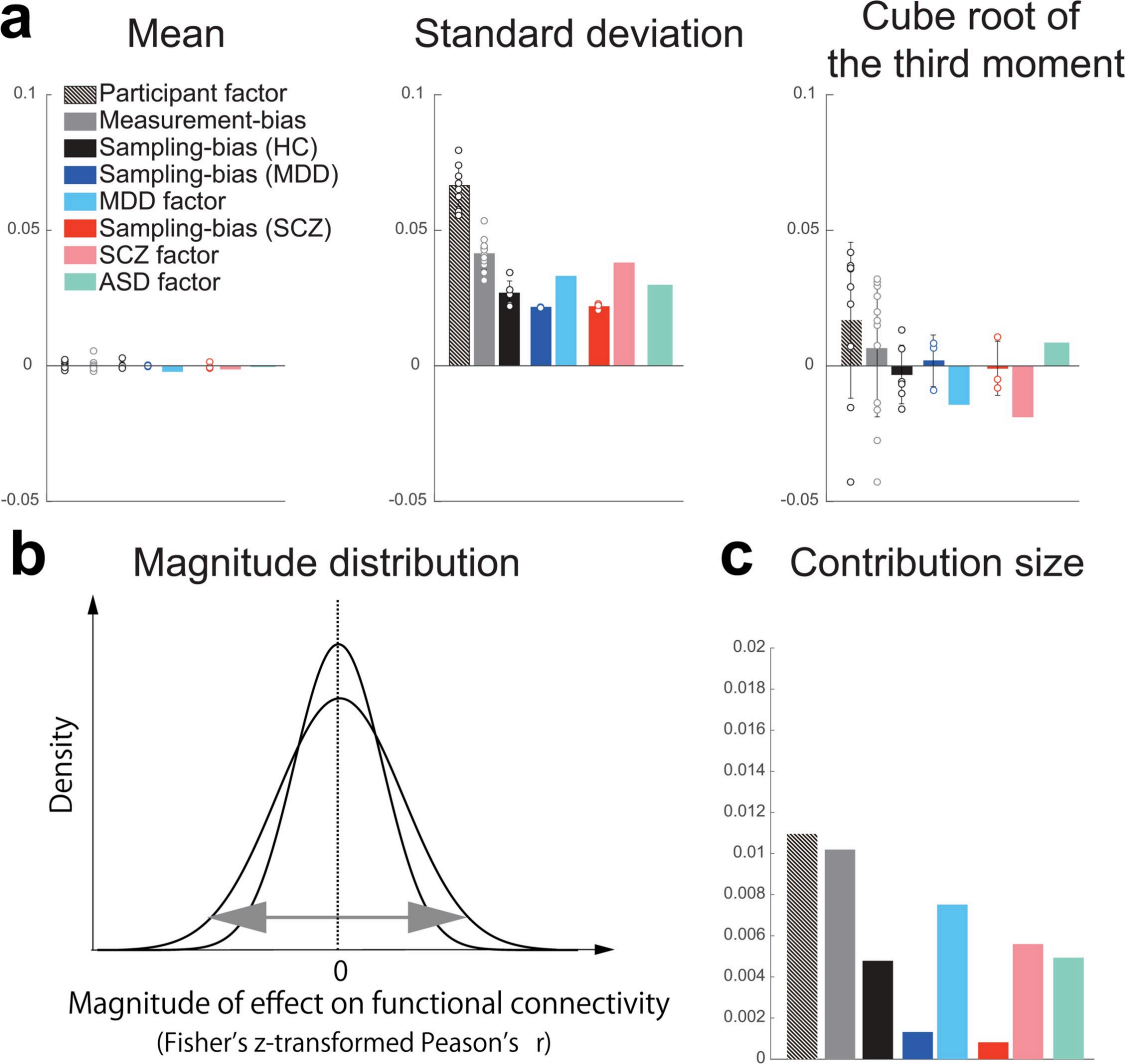
Statistics of magnitude distributions for each type of bias and each factor. (a) The means, standard deviations, and third moments standardized to the same scale on the vertical axis (i.e., cube root) for each type of bias and each factor. Bars represent the average value, and the error bars represent the standard deviation across sites or participants. Each data point represents one participant or one site. (b) Schematic examples illustrating the magnitude distribution. (c) Contribution size of each bias and each factor. The numerical data used in this figure are included in S1 Data. ASD, autism spectrum disorder; HC, healthy control; MDD, major depressive disorder; SCZ, schizophrenia.

To quantitatively evaluate the magnitude of the effect of measurement and sampling biases on functional connectivity, we compared the magnitudes of both types of bias (***m***, ***s***_***hc***_, ***s***_***mdd***_, and ***s***_***scz***_) with the magnitudes of psychiatric disorders (***d***) and participant factors (***p***). For this purpose, we investigated the magnitude distribution of both biases, as well as the effects of psychiatric disorders and participant factors on functional connectivity over all 35,778 elements in a 35,778-dimensional vector (see S1 Text, S1A and S1B Fig). To quantitatively summarize the magnitude of the effect of each factor, we calculated the first, second, and third statistical moments of each distribution (Fig 2A). Based on the mean values and cube roots of the third moments, all distributions could be approximated as bilaterally symmetric with a mean of zero. Thus, distributions with larger squared roots of the second moments (standard deviations) affect more connectivities with larger effect sizes (Fig 2B). The value of the standard deviation was largest for the participant factor (0.0662), followed by these values for the measurement bias (0.0411), the SCZ factor (0.0377), the MDD factor (0.0328), the ASD factor (0.0297), the sampling bias for HCs (0.0267), sampling bias for patients with SCZ (0.0217), and sampling bias for patients with MDD (0.0214). To compare the sizes of the standard deviation of the magnitude distribution between participant factors and measurement bias, we evaluated the variance of each distribution. All pairs of variances were analyzed using Ansari–Bradley tests. Our findings indicated that the variances of magnitude distributions in 10 of 12 measurement biases were significantly larger than in the MDD factor; the variances of magnitude distributions in seven of 12 measurement biases were significantly larger than in the SCZ factor; and the variances of all magnitude distributions in measurement biases were significantly larger than the variance of the MDD factor (S6 Table). The largest variance of magnitude distribution in the sampling bias was significantly larger than in the MDD factor (S7 Table). Variances of magnitude distributions in all participant factors were significantly larger than that in all measurement biases (9 participant factors × 12 measurement biases = 108 pairs; W*: mean = –59.80, maximum = –116.81, minimum = –3.69; *p*-value after Bonferroni correction: maximum = 0.011, minimum = 0, *n* = 35,778). The standard deviation of the magnitude distribution in the participant factor was approximately twice that in the SCZ, MDD, and ASD factors. To investigate similarity in the patterns of effect on functional connectivity between the measurement bias and the disorder factors, we next calculated Pearson’s correlation coefficients between the 12 measurement biases and the factors of three diseases. As a result, we found a significant correlation (mean = 0.13 ± 0.08 [1SD], one-sample *t* test applied to absolute correlation value: *t* = 9.26, *p* < 1.0×10^−10^, *df* = 35), and maximum value was |*r|* = 0.31 (between the MDD factor and the measurement bias of Showa University [SWA]). This result indicates that the pattern of the measurement bias on functional connectivity was not sufficiently different from the patterns of disorder factors in our dataset.

Furthermore, to quantitatively verify the magnitude relationship among factors, we calculated and compared the contribution size to determine the extent to which each bias type and factor explains the variance of the data in our linear model (Fig 2C). After fitting the model, the *b*-th connectivity from subject *a* can be written as follows:

$${Connectivity}_{a,b}={x_{m}^{a}}^{T}m^{b}+{x_{s_{hc}}^{a}}^{T}s_{hc}^{b}+{x_{s_{mdd}}^{a}}^{T}s_{mdd}^{b}+{x_{s_{scz}}^{a}}^{T}s_{scz}^{b}+{x_{d}^{a}}^{T}d^{b}+{x_{p}^{a}}^{T}p^{b}+const+e.$$

For example, the contribution size of measurement bias (i.e., the first term) in this model was calculated as

$${Contribution\;size}_{m}=\frac{1}{N_{m}}\frac{1}{N_{s}*N}\sum_{a=1}^{N_{s}}\sum_{b=1}^{N}\frac{{\left({x_{m}^{a}}^{T}m^{b}\right)}^{2}}{{\left({x_{m}^{a}}^{T}m^{b}\right)}^{2}+{\left({x_{s_{hc}}^{a}}^{T}s_{hc}^{b}\right)}^{2}+{\left({x_{s_{mdd}}^{a}}^{T}s_{mdd}^{b}\right)}^{2}+{\left({x_{s_{scz}}^{a}}^{T}s_{scz}^{b}\right)}^{2}+{\left({x_{d}^{a}}^{T}d^{b}\right)}^{2}+{\left({x_{p}^{a}}^{T}p^{b}\right)}^{2}+e^{2}},$$

in which *N*_*m*_ represents the number of components for each factor, *N* represents the number of connectivities, *N*_*s*_ represents the number of subjects, and *Contribution size*_*m*_ represents the magnitude of the contribution size of measurement bias. This formula was used to assess the contribution sizes of individual factors. The results were consistent with the analysis of the standard deviation (Fig 2A, middle).

These results indicated that the effect size of the measurement bias on functional connectivity is smaller than that of the participant factor but is mostly larger than the disorder factors, which suggested that measurement bias represents a serious limitation in research regarding psychiatric disorders. Furthermore, the effect sizes of the sampling biases were comparable with those of the disorder factors. This finding indicates that sampling bias also represents a major limitation in psychiatric research. In addition, the effect size of the participant factor was much greater than that among patients with SCZ, MDD, or ASD. Such relationships make the development of rs-fcMRI-based classifiers of psychiatric or developmental disorders very challenging. If the disorder factor and site factor are confounded in functional connections, to develop robust and generalizable classifiers across multiple sites, we have to select disorder-specific and site-independent abnormal functional connections [2, 8–10, 15].

### Brain regions contributing most to biases and associated factors

**Fig 3:**
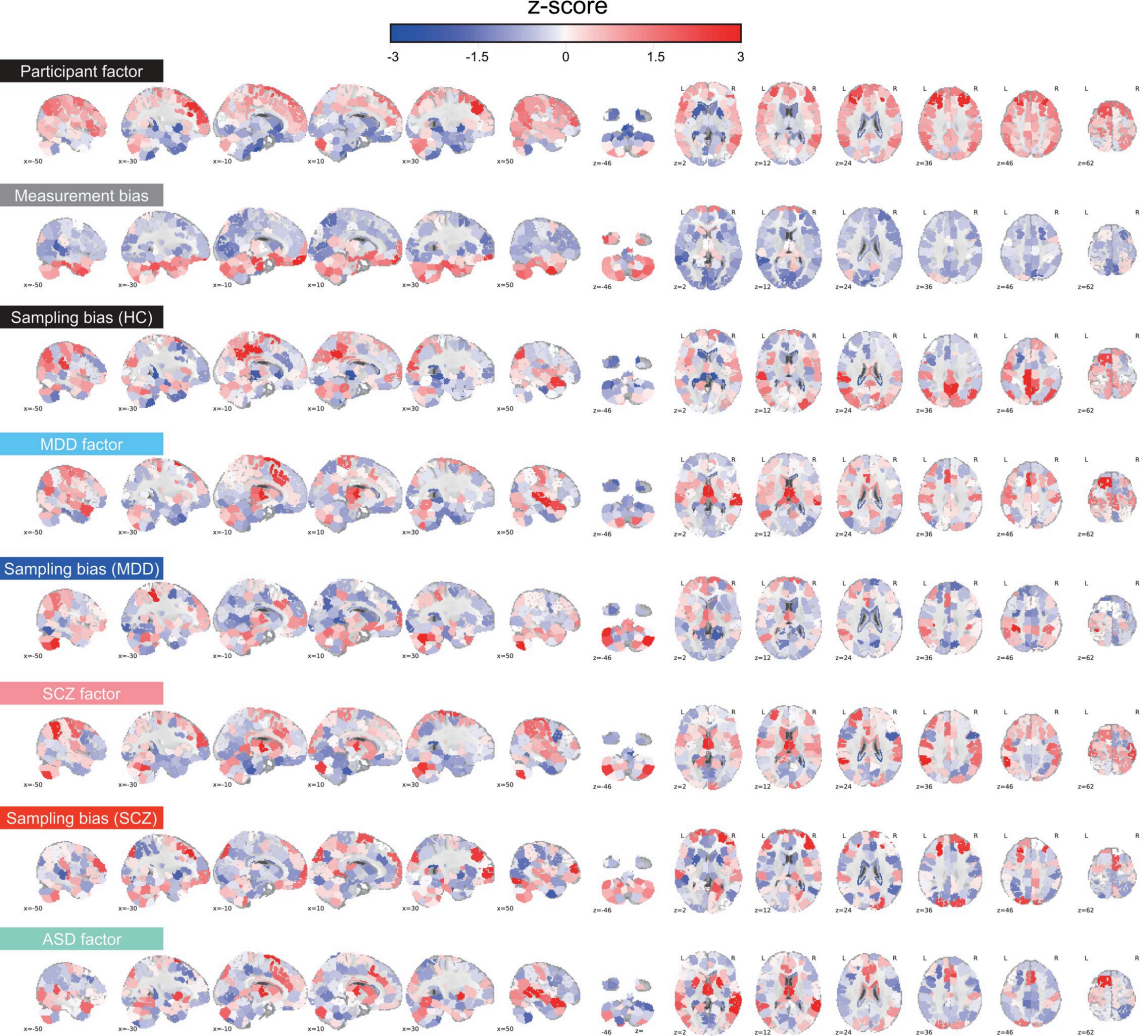
Spatial distribution of each type of bias and each factor in various brain regions. Mean effects of connectivity for all 268 ROIs. For each ROI, the mean effects of all functional connections associated with that ROI were calculated for each bias and each factor. Warmer (red) and cooler (blue) colors correspond to large and small effects, respectively. The magnitudes of the effects are normalized within each bias or each factor (*z*-score). The numerical data used in this figure are included in S1 Data. ASD, autism spectrum disorder; HC, healthy control; MDD, major depressive disorder; ROI, region of interest; SCZ, schizophrenia.

To evaluate the spatial distribution of the two types of bias and all factors in the whole brain, we utilized a previously described visualization method [27] to project connectivity information to anatomical ROIs. First, we quantified the effect of a bias or a factor on each functional connectivity as the median of its absolute values across sites or participants. Thus, we obtained 35,778 values, each of which was associated with one connectivity and represented the effect of a bias or factor on the connectivity. Next, we summarized these effects on connectivity for each ROI by averaging the values of all connectivities connected with the ROI (see “Spatial characteristics of measurement bias, sampling bias, and each factor in the brain” in the Methods section). The average value represents the extent the ROI contributes to the effect of a bias or factor. By repeating this procedure for each ROI and coding the averaged value based on the color of an ROI, we were able to visualize the relative contribution of individual ROIs to each bias or factor in the whole brain (Fig 3). Consistent with the findings of previous studies, the effect of the participant factor was large for several ROIs in the cerebral cortex, especially in the prefrontal cortex, but small in the cerebellum and visual cortex [24]. The effect of measurement bias was large in inferior brain regions where functional images are differentially distorted depending on the phase-encoding direction [28, 29]. Connections involving the medial dorsal nucleus of the thalamus were also heavily affected by both MDD, SCZ, and ASD. Effects of the MDD factor were observed in the dorsomedial prefrontal cortex and superior temporal gyrus, in which abnormalities have also been reported in previous studies [22, 30, 31]. Effects of the SCZ factor were observed in the left inferior parietal lobule, bilateral anterior cingulate cortices, and left middle frontal gyrus, in which abnormalities have been reported in previous studies [32–34]. Effects of the ASD factor were observed in the putamen, the medial prefrontal cortex, and the right middle temporal gyrus, in which abnormalities have also been reported in previous studies [10, 11, 35]. The effect of sampling bias for HCs was large in the inferior parietal lobule and the precuneus, both of which are involved in the default-mode network and the middle frontal gyrus. Sampling bias for disorders was large in the medial dorsal nucleus of the thalamus, left dorsolateral prefrontal cortex, dorsomedial prefrontal cortex, and cerebellum for MDD [22] and in the prefrontal cortex, cuneus, and cerebellum for SCZ [33].

### Characteristics of measurement bias

**Fig 4:**
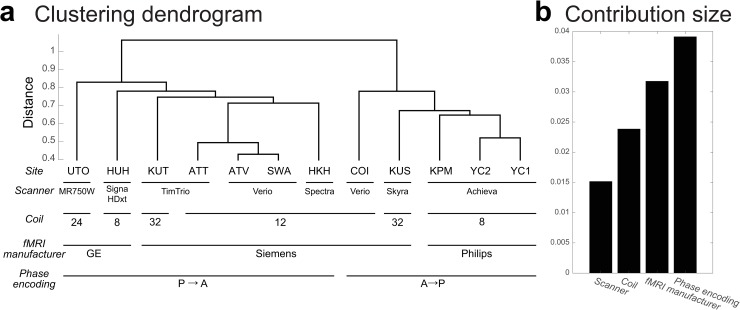
Clustering dendrogram for measurement bias. (a) The height of each linkage in the dendrogram represents the dissimilarity (1 − *r*) between the clusters joined by that link. (b) Contribution size of each factor. The numerical data used in this figure are included in S1 Data. ATT, Siemens TimTrio scanner at Advanced Telecommunications Research Institute International; ATV, Siemens Verio scanner at Advanced Telecommunications Research Institute International; COI, Center of Innovation in Hiroshima University; fMRI, functional magnetic resonance imaging; HKH, Hiroshima Kajikawa Hospital; HUH, Hiroshima University Hospital; KPM, Kyoto Prefectural University of Medicine; KUS, Siemens Skyra scanner at Kyoto University; KUT, Siemens TimTrio scanner at Kyoto University; SWA, Showa University; UTO, University of Tokyo; YC1, Yaesu Clinic 1; YC2, Yaesu Clinic 2.

We next investigated the characteristics of measurement bias. We first examined whether similarities among the estimated measurement bias vectors for the 12 included sites reflect certain properties of MRI scanners such as phase-encoding direction, MRI manufacturer, coil type, and scanner type. We used hierarchical clustering analysis to discover clusters of similar patterns for measurement bias. We used “correlation” as a distance metric for hierarchical clustering. This method has previously been used to distinguish subtypes of MDD, based on rs-fcMRI data [22]. As a result, the measurement biases of the 12 sites were divided into phase-encoding direction clusters at the first level (Fig 4A). They were divided into fMRI manufacturer clusters at the second level and further divided into coil type clusters, followed by scanner model clusters. Furthermore, we quantitatively verified the relationship magnitude among factors by using the same model to assess the contribution of each factor (Fig 4B; see “Quantification of site differences” in the Results section or “Analysis of contribution size” in the Methods section). The contribution size was largest for the phase-encoding direction (0.0391), followed by the contribution sized for fMRI manufacturer (0.0318), coil type (0.0239), and scanner model (0.0152). These findings indicate that the main factor influencing measurement bias is the difference in the phase-encoding direction, followed by fMRI manufacturer, coil type, and scanner model, respectively.

### Sampling bias is because of sampling from among a subpopulation

**Fig 5:**
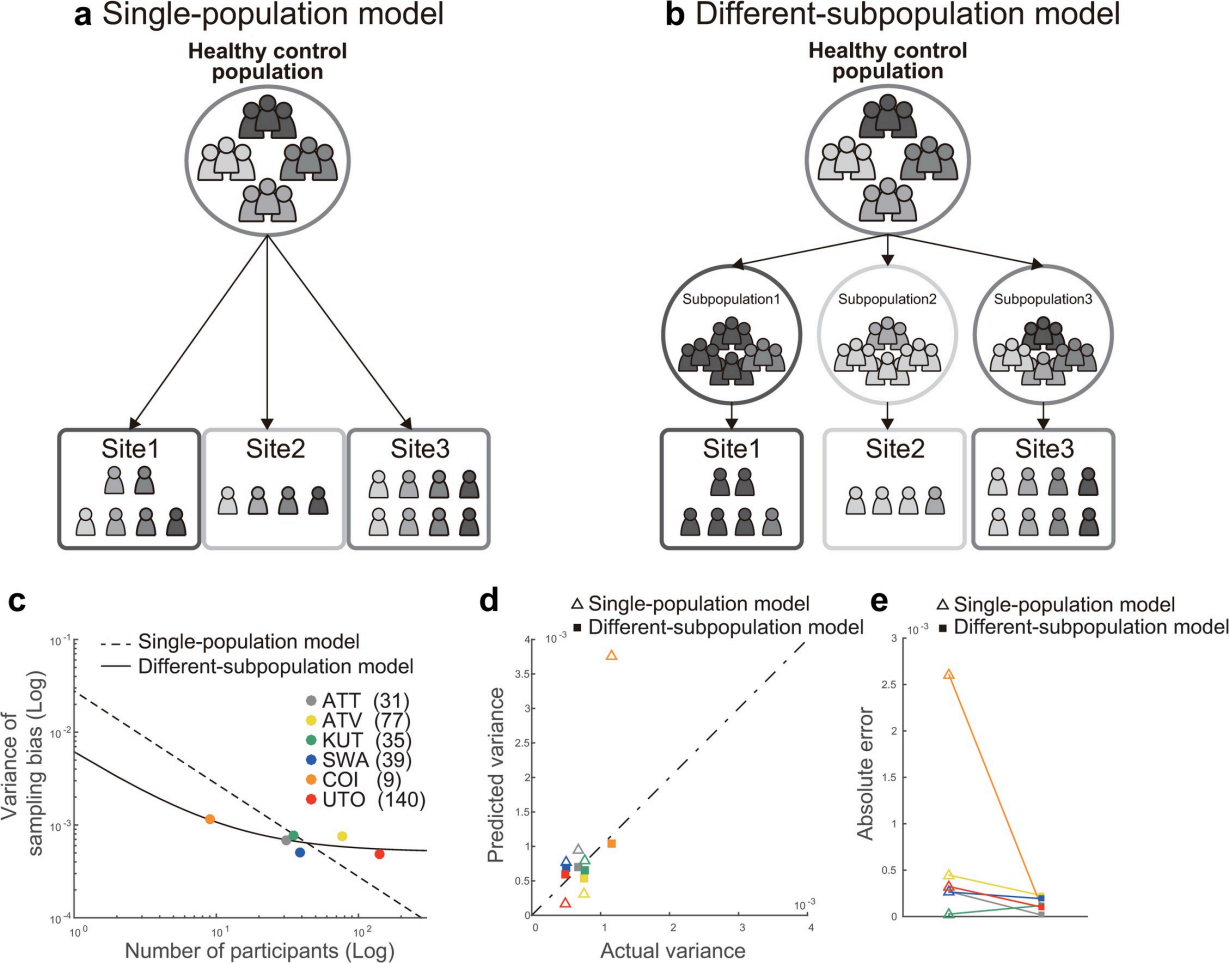
Comparison of the two models of sampling bias. Schematic examples illustrating the (a) Single-population and (b) Different-subpopulation models and (c) The results of model fitting. The *x* axis represents the number of participants on a logarithmic scale, and the *y* axis represents the variance of sampling bias on a logarithmic scale. The broken line represents the prediction of the single-population model, whereas the solid line represents the prediction of the different-subpopulation model. Each data point represents one site. (d) Results of the predictions determined by using each model. The *x* axis represents the actual variance, and the *y* axis represents the predicted variance. Open triangles correspond to the single-population model, whereas filled squares correspond to the different-subpopulation model. (e) Performance of prediction using the two models, based on the absolute error between the actual and predicted variance. The numerical data used in this figure are included in S1 Data. ATT, Siemens TimTrio scanner at Advanced Telecommunications Research Institute International; ATV, Siemens Verio scanner at Advanced Telecommunications Research Institute International; COI, Center of Innovation in Hiroshima University; KUT, Siemens TimTrio scanner at Kyoto University; SWA, Showa University; UTO, University of Tokyo.

We investigated two alternative models for the mechanisms underlying sampling bias. In the single-population model, which assumes that participants are sampled from a common population (Fig 5A), the functional connectivity values of each participant were generated from a Gaussian distribution, with a mean of 0 and variance of *ξ*^2^ for each connectivity value. Then, the functional connectivity vector for participant *j* at site *k* can be described as

$$c_{j}^{k}∼N(0,\xi^{2}I).$$

In the different-subpopulation model, which assumes that sampling bias occurs partly because participants are sampled from different subpopulations at each site (Fig 5B), we assumed that the functional connectivity values of each participant were generated from a different independent Gaussian distribution, with an average of ***β***_***k***_ and a variance of *ξ*^2^ depending on the population of each site. In this situation, the functional connectivity vector for participant *j* at site *k* can be described as

$$c_{j}^{k}∼N(\beta_{k},\xi^{2}I).$$

Here, we assume that the average of the population ***β***_***k***_ is sampled from an independent Gaussian distribution with an average of 0 and a variance of *σ*^2^.

It is necessary to determine which model is more suitable for collecting big data across multiple sites: If the former model is correct, then the data can be used to represent a population by increasing the number of participants, even if the number of sites is small. If the latter model is correct, data should be collected from many sites, as a single site does not represent the true grand population distribution, even with a very large sample size.

For each model, we first investigated how the number of participants at each site determined the effect of sampling bias on functional connectivity. We measured the magnitude of the effect, based on the variance values for sampling bias across functional connectivity (see the “Quantification of site differences” section). We used variance instead of the standard deviation to simplify the statistical analysis, although there is essentially no difference based on which value is used. We theorized that each model represents a different relationship between the number of participants and the variance of sampling bias. Therefore, we investigated which model best represents the actual relationships in our data by comparing the corrected Akaike information criterion (AICc) [36, 37] and Bayesian information criterion (BIC). Moreover, we performed leave-one-site-out cross-validation evaluations of predictive performance in which all but one site was used to construct the model, and the variance of the sampling bias was predicted for the remaining site. We then compared the predictive performances between the two models. Our results indicated that the different-subpopulation model provided a better fit for our data than the single-population model (Fig 5C; different-subpopulation model: AICc = –108.80 and BIC = –113.22; single-population model: AICc = –96.71 and BIC = –97.92). Furthermore, the predictive performance was significantly higher for the different-subpopulation model than for the single-population model (one-tailed Wilcoxon signed rank test applied to absolute errors: Z = 1.67, *p* = .0469, *n* = 6; Fig 5D and 5E). This result indicates that sampling bias is caused not only by random sampling from a single grand population, depending on the number of participants among sites, but also by sampling from among different subpopulations. Sampling biases thus represent a major limitation in attempting to estimate a true single distribution of HC or patient data based on measurements obtained from a finite number of sites and participants.

### Visualization of the effect of harmonization

Since our results indicated that the patterns of the measurement bias on functional connectivity were not sufficiently different from the patterns of disorder factors, we need harmonization to properly subtract the measurement bias. Therefore, we next developed a novel harmonization method that enabled us to subtract measurement bias alone using the traveling-subject dataset. Using a constrained linear regression model, we estimated measurement bias separately from sampling bias (see “Bias estimation” in the Methods section). Thus, we could remove the measurement bias from the SRPBS multidisorder dataset (i.e., traveling-subject method, see “Traveling-subject harmonization” in the Methods section).

**Fig 6:**
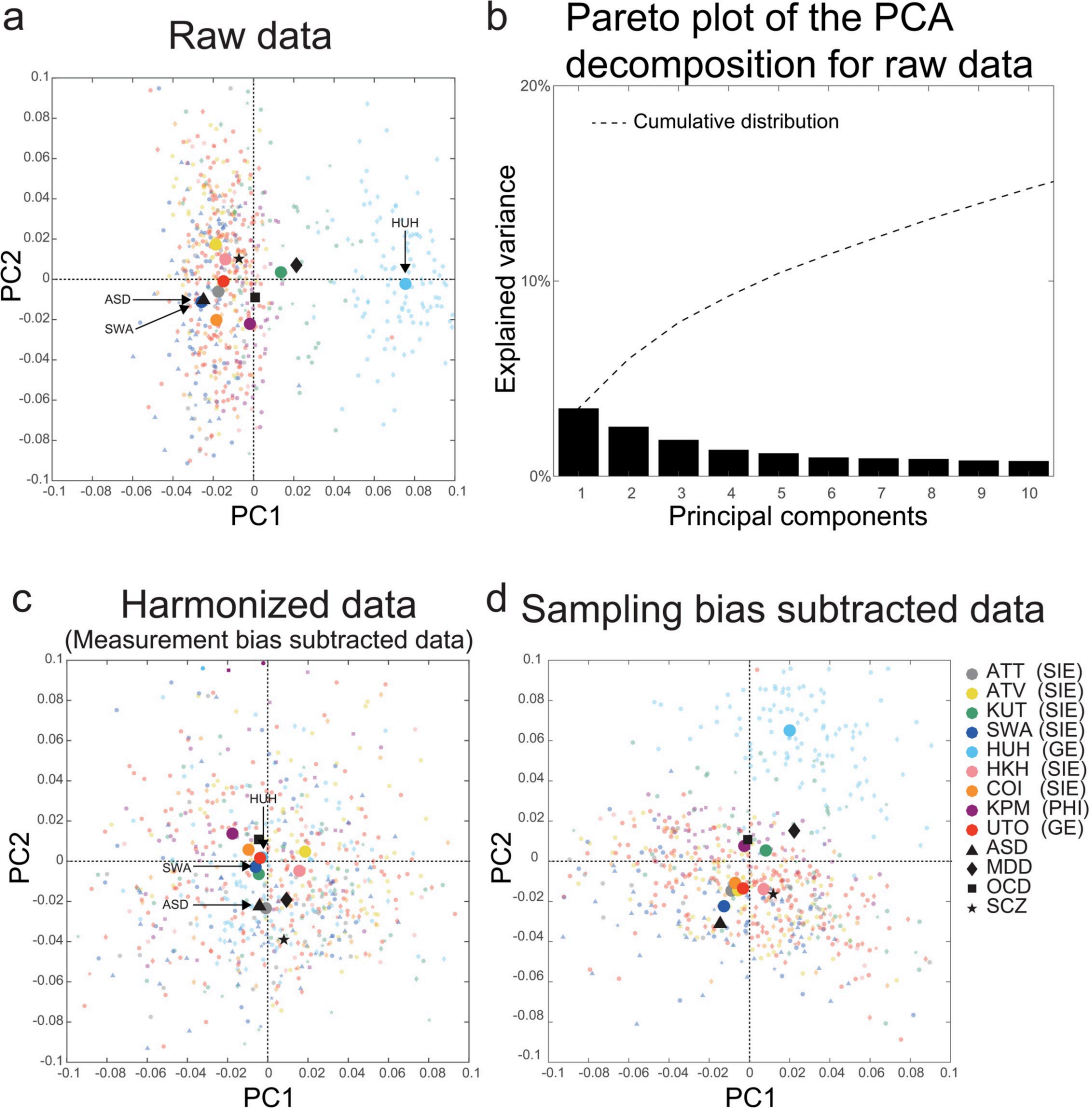
PCA dimension reduction in the SRPBS multidisorder dataset. Comparison among (a) Raw data, (c) Harmonized data (measurement bias subtracted data), and (d) Sampling bias subtracted data. All participants in the SRPBS multidisorder dataset projected into the first two PCs, as indicated by small, light-colored markers. Dark-colored markers indicate the averages of the projected data across healthy controls at each site and the average within each psychiatric disorder in the subspace spanned by the two components. The color of the marker represents the site, whereas the shape represents the psychiatric disorder. (b) The pareto plot of the PCA decomposition for raw data. The pareto plot shows how much variance is explained by each principal component. The numerical data used in this figure are included in S1 Data. ASD, autism spectrum disorder; ATT, Siemens TimTrio scanner at Advanced Telecommunications Research Institute International; ATV, Siemens Verio scanner at Advanced Telecommunications Research Institute International; COI, Center of Innovation in Hiroshima University; GE, GE functional magnetic resonance imaging; HKH, Hiroshima Kajikawa Hospital; HUH, Hiroshima University Hospital; KPM, Kyoto Prefectural University of Medicine; KUT, Siemens TimTrio scanner at Kyoto University; MDD, major depressive disorder; OCD, obsessive compulsive disorder; PC, principal component; PCA, PC analysis; PHI, Philips functional magnetic resonance imaging; SCZ, schizophrenia; SIE, Siemens functional magnetic resonance imaging; SRPBS, Strategic Research Program for Brain Sciences; SWA, Showa University; UTO, University of Tokyo.

To visualize the site differences and disorder effects in the SRPBS multidisorder dataset while maintaining its quantitative properties, we first performed an unsupervised dimension reduction of the raw rs-fcMRI data using a principal component analysis (PCA). All participant data in the SRPBS multidisorder dataset were plotted on two axes consisting of the first two principal components (PCs) (Fig 6A, small, light-colored symbols). The first two PCs could explain approximately 6% of the variance in the whole data (Fig 6B, 3.5% and 2.5% for the first and second PC, respectively). Dark-colored markers indicated the averages of projected data across HCs in each site and the average within each psychiatric disorder in the subspace spanned by the two components. For the raw data, there was a clear separation of the Hiroshima University Hospital (HUH) site for PC1, which explained most of the variance in the data. To visualize the effects of the harmonization process, we plotted the data after subtracting only the measurement bias from the SRPBS multidisorder dataset (Fig 6C). In Fig 6C, the differences among sites represent the sampling bias. Relative to the result of raw data, which reflects the data before harmonization, the HUH site moved much closer to the origin (i.e., grand average) and showed no marked separation from the other sites. This result indicated that the separation of the HUH site observed in Fig 6A was caused by measurement bias, which was removed by the harmonization. Furthermore, harmonization was effective in distinguishing patients and HCs scanned at the same site. Since patients with ASD were only scanned at the SWA site, the averages for these patients (▲) and HCs (blue ●) scanned at this site were projected to nearly identical positions (Fig 6A). However, the two symbols are clearly separated from one another in Fig 6C. The effect of a psychiatric disorder (ASD) could not be observed in the first two PCs without harmonization but became detectable following the removal of measurement bias. Finally, to visualize the measurement bias in the SRPBS multidisorder dataset, we plotted the data after subtracting only the sampling bias from the SRPBS multidisorder dataset (Fig 6D). Relative to the harmonized data results, the HUH site showed marked separation from the other sites, which was similar to the raw data (Fig 6A).

### Quantification of the effect of traveling-subject harmonization

To correct difference among sites, there are three commonly used harmonization methods: (1) a ComBat method [16, 17, 19, 38], a batch-effect correction tool commonly used in genomics, site difference was modeled and removed; (2) a generalized linear model (GLM) method, site difference was estimated without adjusting for biological covariates (diagnosis) [16, 18, 22]; and (3) an adjusted GLM method, site difference was estimated while adjusting for biological covariates [16, 18] (see “Harmonization procedures” in the Methods section). However, all these methods estimate site difference without separating it into measurement and sampling biases and subtracting the site difference from the data. Therefore, existing harmonization methods might have pitfalls that eliminate both biologically meaningless measurement bias and biologically meaningful sampling bias. Here, we tested whether the traveling-subject harmonization method indeed removes only the measurement bias and whether the existing harmonization methods simultaneously remove the measurement and sampling biases. Specifically, we performed 2-fold cross-validation evaluations in which the SRPBS multidisorder dataset was partitioned into two equal-size subsamples (fold1 data and fold2 data) with the same proportions of sites. Between these two subsamples, the measurement bias is common, but the sampling bias is different, because the scanners are common and participants are different. We estimated the measurement bias (or site difference including the measurement bias and the sampling bias for the existing methods) by applying the harmonization methods to the fold1 data and subtracting the measurement bias or site difference from the fold2 data. Next, we estimated the measurement bias in the fold2 data. For the existing harmonization methods, if the site difference estimated using fold1 contained only the measurement bias, the measurement bias estimated in fold2 data after subtracting the site difference should be smaller than without subtracting the site difference (Raw). To separately estimate measurement bias and sampling bias in both subsamples while avoiding information leaks, we also divided the traveling-subject dataset into two equal-size subsamples with the same proportions of sites and subjects. We concatenated one subsample of traveling-subject dataset to fold1 data to estimate the measurement bias for traveling-subject method (estimating dataset) and concatenated the other subsample of traveling-subject dataset to fold2 data for testing (testing dataset). That is, in the traveling-subject harmonization method, we estimated the measurement bias using the estimating dataset and removed the measurement bias from the testing dataset. By contrast, in the other harmonization methods, we estimated the site difference using the fold1 data (not including the subsample of traveling-subject dataset) and removed the site difference from the testing dataset. We then estimated the measurement bias using the testing dataset and evaluated the standard deviation of the magnitude distribution of measurement bias calculated in the same way as described in the “Quantification of site differences” section. To verify whether important information, such as participant and disorder factors, remained in the testing dataset, we also estimated these factors and calculated the ratio of the standard deviation of the magnitude distribution of the measurement bias to each participant and disorder factor as signal-to-noise ratios. This procedure was performed again by exchanging the estimating dataset and the testing dataset (see “Twofold cross-validation evaluation procedure” in the Methods section).

**Fig 7:**
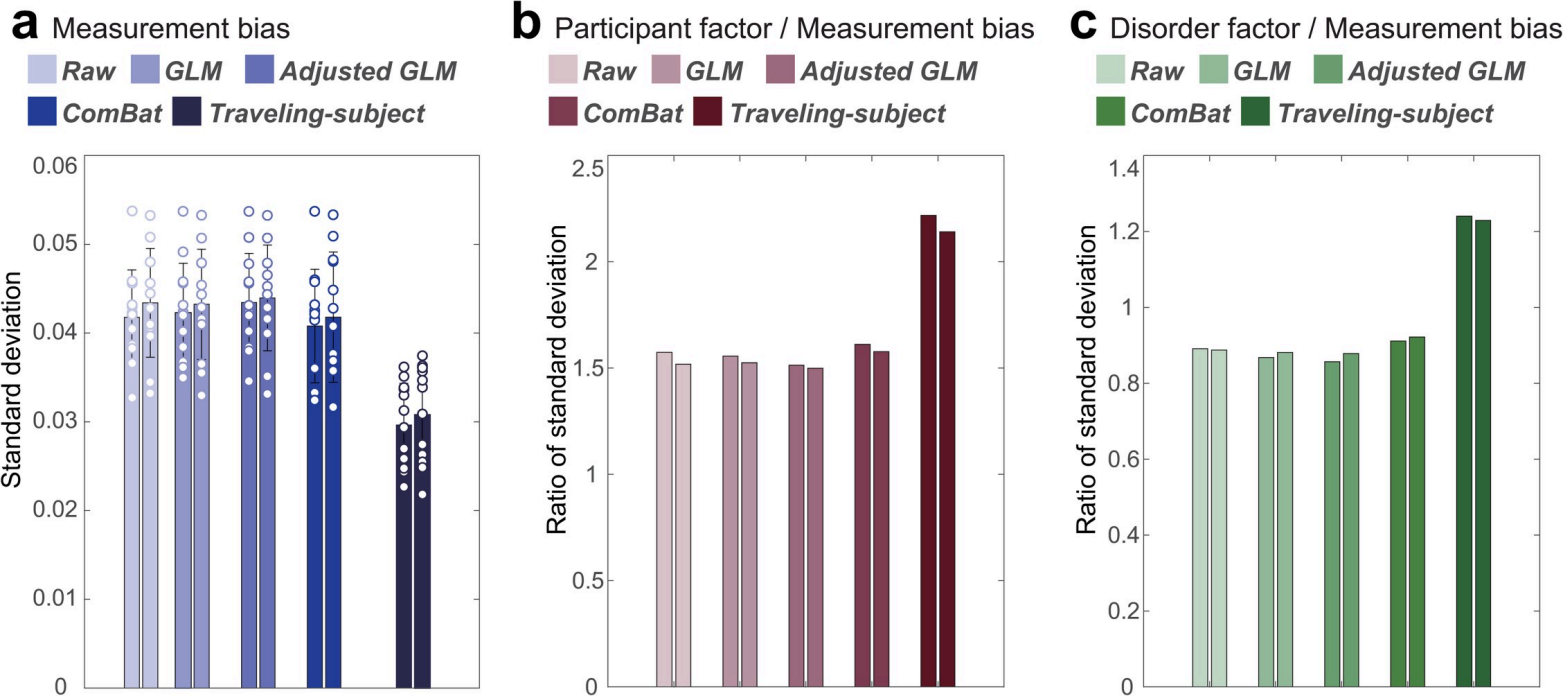
Reduction of the measurement bias and improvement of signal-to-noise ratios for different harmonization methods. (a) Standard deviation of the magnitude distribution of the measurement bias. The error bars represent the standard deviation across sites. Each data point represents one site. (b) Ratio of standard deviation of the magnitude distribution of the measurement bias to that of the participant factor. (c) Ratio of standard deviation of the magnitude distribution of the measurement bias to that of the disorder factor. Different colored columns show the results from different harmonization methods. Two columns of the same color show the results of the two folds. The numerical data used in this figure are included in S1 Data. GLM, generalized linear model.

Fig 7 shows the standard deviation of the magnitude distribution of the measurement bias and the ratio of the standard deviation of the magnitude distribution of the measurement bias to that of participant factor and disorder factor in both fold data for the four harmonization methods and without harmonization (Raw). Our results show the highest reduction of the standard deviation of the magnitude distribution of the measurement bias from the Raw in the traveling-subject method when compared with all other methods (29% versus 3% in the ComBat method). Moreover, improvements in the signal-to-noise ratios were also highest in our method for the participant factor (41% versus 3% in the ComBat method) and disorder factor (39% versus 3% in the ComBat method). These results indicated that the traveling-subject harmonization method removed measurement bias and improved the signal-to-noise ratios.

## Discussion

In the present study, by acquiring a separate traveling-subject dataset and the SRPBS multidisorder dataset, we separately estimated measurement and sampling biases for multiple sites, which we then compared with the magnitude of disorder factors. Furthermore, we investigated the origin of each bias in multisite datasets. Finally, to overcome the problem of site differences, we developed a novel harmonization method that enabled us to subtract the measurement bias by using a traveling-subject dataset and achieved the reduction of the measurement bias by 29% and improvement of signal-to-noise ratios by 40%.

Previous studies have focused on measurement bias and compared its magnitude to the participant factor by using a traveling-subject design in a finger-tapping task fMRI [39] and rs-fMRI [20]. These studies revealed the magnitude of measurement bias is smaller than the participant factor. Although such a result was also obtained in this study, the novelty of this study exists in that we separately estimated measurement and sampling biases and then compared them with the magnitude of disorder factors. Our findings indicated that measurement bias exerted significantly greater effects than disorder factors, whereas sampling bias was comparable with (or even larger than) the disorder effects (Fig 2). Although our effect-size analysis was univariate, it is important to take perspective of multivariate pattern analysis into account because biomarker construction is based on the multivariate pattern of functional connectivity. From this view point, if the effect of the measurement bias is orthogonal to that of the psychiatric disorders, robust generalization across sites might be possible. Actually, previous research suggested this [11]. However, the orthogonality between the pattern of the disease factors and that of the measurement bias depends on dataset and type of disease. Since our results indicated that the pattern of the measurement bias was not sufficiently different from the patterns of disorder factors, harmonization was important to properly subtract the measurement bias. This result is a very important finding for future studies that collect rs-fMRI data from multiple sites and for consideration when constructing biomarkers of psychiatric disorders based on multisite data in the clinical field. Our results indicate that it is important to consider site differences when we investigate disorder factors using multisite rs-fMRI data. However, we did not control for variations in disease stage and treatment in our dataset. Although controlling for such heterogeneity may increase the effect size of disorder factors, such control is not feasible when collecting big data from multiple sites. Therefore, it is important to appropriately remove measurement bias from heterogeneous patient data to identify relatively small disorder effects. This issue is essential for investigating the relationships among different psychiatric disorders because disease factors could be confounded by site differences. As previously mentioned, it is common for a single site to sample only a few types of psychiatric disorders (SCZ and ASD from sites A and B, respectively). This issue is also essential for constructing biomarkers of psychiatric disorders because classification of subjects can be achieved by using the information of nonbiological site difference. In this situation, it is critical to dissociate disease factors from site differences. This dissociation can be accomplished by subtracting only the measurement bias, which is estimated using the traveling-subject dataset.

Our results indicated that measurement bias is primarily influenced by differences in the phase-encoding direction, followed by differences in fMRI manufacturer, coil type, and scanner model (Fig 4). These results are consistent with our finding of large measurement biases in the inferior brain regions (Fig 3), the functional imaging of which is known to be influenced by the phase-encoding direction [28, 29]. Previous studies have reported that the effect caused by the difference in the phase-encoding direction can be corrected using the field map obtained at the time of imaging [28, 40–42]. The field map was acquired in parts of the traveling-subject dataset; therefore, we investigated the effectiveness of field map correction by comparing the effect size of the measurement bias and the participant factor between functional images with and without field map correction (see S2 Text). Our prediction was as follows: if field map correction is effective, the effect of measurement bias will decrease, whereas that of the participant factor will increase following field map correction. Field map correction using SPM12 (http://www.fil.ion.ucl.ac.uk/spm/software/spm12) reduced the effect of measurement bias in the inferior brain regions (whole brain: 3% reduction in the standard deviation of the magnitude distribution of the measurement bias) and increased the effect of the participant factor in the whole brain (3% increase in the standard deviation of the magnitude distribution of the participant factor; S2A and S2B Fig). However, the effect of measurement bias remained large in inferior brain regions (S2A Fig), and hierarchical clustering analysis revealed that the clusters of the phase-encoding direction remained dominant (S2C Fig). These results indicate that, even with field map correction, it is largely impossible to remove the influence of differences in phase-encoding direction on functional connectivity. Thus, harmonization methods are still necessary to remove the effect of these differences and other measurement-related factors. However, some distortion correction methods have been developed, such as the top-up method and symmetric normalization [43, 44], and further studies are required to verify the efficacy of these methods.

Our data supported the different-subpopulation model rather than the single-population model (Fig 5), which indicates that sampling bias is caused by sampling from among different subpopulations. Furthermore, these findings suggest that, during big data collection, it is better to sample participants from several imaging sites than to sample many participants from a few imaging sites. These results were obtained only by combining the SRPBS multidisorder database with a traveling-subject dataset (https://bicr.atr.jp/decnefpro/). To the best of our knowledge, the present study is the first to demonstrate the presence of sampling bias in rs-fcMRI data, the mechanisms underlying this sampling bias, and the effect size of sampling bias on resting-state functional connectivity, which was comparable to that of psychiatric disorders. We analyzed sampling bias among HCs only, because the number of sites was too small to conduct an analysis of patients with psychiatric diseases.

We developed a novel harmonization method using a traveling-subject dataset (i.e., traveling-subject method), which was then compared with existing harmonization methods. Our results demonstrated that the traveling-subject method outperformed other conventional GLM-based harmonization methods and the ComBat method. The traveling-subject method achieved reduction in the measurement bias by 29%, compared with 3% in the second highest value for the ComBat method, and improvement in the signal-to-noise ratios by 40%, compared with 3% in the second highest value for the ComBat method. This result indicates that the traveling-subject dataset helps us to properly estimate measurement bias and harmonize the rs-fMRI data across imaging sites toward development of a wide range of final applications. As one example of such applications, we constructed biomarkers for psychiatric disorders based on rs-fcMRI data, which distinguishes between HCs and patients, and a regression model to predict participants’ age based on rs-fcMRI data using the SRPBS multidisorder dataset (see “Classifiers for MDD and SCZ, based on the four harmonization methods” in S5 Text and “Regression models of participant age based on the four harmonization methods” in S6 Text). We quantitatively evaluated the harmonization method to investigate the generalization performance to independent validation dataset, which was not included in the SRPBS multidisorder dataset. Although the ComBat method achieved the highest performance for the MDD classifier and regression model of age, it was inferior to the raw method for the SCZ classifier. By contrast, the traveling-subject harmonization method always improved the generalization performance compared with when no harmonization was performed. This result also indicates that the pattern of the measurement bias on functional connectivity was not sufficiently different from the patterns of disorder factors in our dataset. These results indicate that the traveling-subject dataset also helps with constructing a prediction model based on multisite rs-fMRI data. Future work should improve the traveling-subject method by incorporating a hierarchical model, such as ComBat.

The present study possesses some limitations of note. The accuracy of measurement bias estimation may be improved by further expanding the traveling-subject dataset. This can be achieved by increasing the number of traveling participants or sessions per site. However, as mentioned in a previous traveling-subject study [20], it is costly and time-consuming to ensure that numerous participants travel to every site involved in big database projects. Thus, the cost-performance tradeoff must be evaluated in practical settings. The numbers of traveling participants and MRI sites used in this study (9 and 12, respectively) were larger than those used in a previous study (8 and 8, respectively) [20], and the number of total sessions in this study (411) was more than three times larger than that used in the previous study (128) [20]. Furthermore, although we estimated the measurement bias for each connectivity, hierarchical models of the brain (e.g., ComBat) may be more appropriate for improving the estimates of measurement bias. Regarding the number of sites in the data with psychiatric disorders, we believe that uniqueness of our study exists in the datasets of multiple disorders and multiple sites with traveling-subject data rather than the number of sites for a single disorder. For example, although ABIDE [6, 11] collected the data from patients with ASD from 17 sites, it significantly differs from our study because it does not use a unified protocol for data collection and does not include a traveling-subject dataset. In this study, we have collected the data using a unified protocol with HCs from six sites, patients with MDD from three sites, patients with ASD from one site, patients with SCZ from three sites, patients with OCD from one site, and a traveling-subject dataset from 12 sites. These datasets enabled us to compare the magnitude of the effect between site differences (measurement or sampling bias) and multiple disorder factors, which is the key point of our study. To the best of our knowledge, such a multisite, multidisorder rs-fMRI dataset has not existed so far.

In summary, by acquiring a separate traveling-subject dataset and the SRPBS multidisorder database, we revealed that site differences were composed of biological sampling bias and engineering measurement bias. The effect sizes of these biases on functional connectivity were greater than or equal to the effect sizes of psychiatric disorders, and the pattern of the measurement bias was not sufficiently different from the patterns of disorder factors, highlighting the importance of controlling for site differences when investigating psychiatric disorders. Furthermore, using the traveling-subject dataset, we developed a novel traveling-subject method that harmonizes the measurement bias only by separating sampling bias from site differences. Our findings verified that the traveling-subject method outperformed conventional GLM-based harmonization methods and ComBat method. These results suggest that a traveling-subject dataset can help to harmonize the rs-fMRI data across imaging sites.

## Methods

### Ethics statement

All participants in all datasets provided written informed consent. All recruitment procedures and experimental protocols were approved by the institutional review boards of the principal investigators’ respective institutions (Advanced Telecommunications Research Institute International [ATR] [approval numbers: 13–133, 14–133, 15–133, 16–133, 17–133, and 18–133], Hiroshima University [E-38], Kyoto Prefectural University of Medicine [KPM] [RBMR-C-1098], SWA [B-2014-019 and UMIN000016134], the University of Tokyo [UTO] Faculty of Medicine [3150], Kyoto University [C809 and R0027], and Yamaguchi University [H23-153 and H25-85]) and conducted in accordance with the Declaration of Helsinki.

### Participants

We used two rs-fMRI datasets for all analyses: (1) the SRPBS multidisorder dataset, which encompasses multiple psychiatric disorders, and (2) a traveling-subject dataset. The SRPBS multidisorder dataset contains data for 805 participants (482 HCs from nine sites, 161 patients with MDD from five sites, 49 patients with ASD from one site, 65 patients with OCD from one site, and 48 patients with SCZ from three sites) (Table 1). Data were acquired using a Siemens TimTrio scanner at ATR (ATT), a Siemens Verio scanner at ATR (ATV), a Siemens Verio at the Center of Innovation in Hiroshima University (COI), a GE SignaHDxt scanner at HUH, a Siemens Spectra scanner at Hiroshima Kajikawa Hospital (HKH), a Philips Achieva scanner at KPM, a Siemens Verio scanner at SWA, a Siemens TimTrio scanner at Kyoto University (KUT), and a GE MR750W scanner at the UTO. Each participant underwent a single rs-fMRI session for 5–10 min. The rs-fMRI data were acquired using a unified imaging protocol at all but three sites (Table 2; https://bicr.atr.jp/rs-fmri-protocol-2/). During the rs-fMRI scans, participants were instructed as follows, except at one site: “Please relax. Don’t sleep. Fixate on the central crosshair mark and do not think about specific things.” At the remaining site, participants were instructed to close their eyes rather than fixate on a central crosshair.

In the traveling-subject dataset, nine healthy participants (all male participants; age range 24–32 y; mean age 27 ± 2.6 y) were scanned at each of 12 sites in the SRPBS consortium, producing a total of 411 scan sessions. Data were acquired at the sites included in the SRPBS multidisorder database (i.e., ATT, ATV, COI, HUH, HKH, KPM, SWA, KUT, and UTO) and three additional sites: Kyoto University (KUS; Siemens Skyra) and Yaesu Clinic 1 and 2 (YC1 and YC2; Philips Achieva) (S1 Table). Each participant underwent three rs-fMRI sessions of 10 min each at nine sites, two sessions of 10 min each at two sites (HKH and HUH), and five cycles (morning, afternoon, next day, next week, and next month) consisting of three 10-min sessions each at a single site (ATT). In the latter situation, one participant underwent four rather than five sessions at the ATT site because of a poor physical condition. Thus, a total of 411 sessions were conducted [8 participants × (3 × 9 + 2 × 2 + 5 × 3 × 1) + 1 participant × (3 × 9 + 2 × 2 + 4 × 3 × 1)]. During each rs-fMRI session, participants were instructed to maintain a focus on a fixation point at the center of a screen, remain still and awake, and to think about nothing in particular. For sites that could not use a screen in conjunction with fMRI (HKH and KUS), a seal indicating the fixation point was placed on the inside wall of the MRI gantry. Although we attempted to ensure imaging was performed using the same variables at all sites, there were two phase-encoding directions (P→A and A→P), three MRI manufacturers (Siemens, GE, and Philips), four different numbers of channels per coil (8, 12, 24, and 32), and seven scanner types (TimTrio, Verio, Skyra, Spectra, MR750W, SignaHDxt, and Achieva) (S1 Table).

### Preprocessing and calculation of the resting-state functional connectivity matrix

The rs-fMRI data were preprocessed using SPM8 implemented in MATLAB (R2016b; Mathworks, Natick, MA, USA). The first 10 s of data was discarded to allow for T1 equilibration. Preprocessing steps included slice-timing correction, realignment, coregistration, segmentation of T1-weighted structural images, normalization to Montreal Neurological Institute (MNI) space, and spatial smoothing with an isotropic Gaussian kernel of 6 mm full-width at half-maximum. For the analysis of connectivity matrices, ROIs were delineated according to a 268-node gray matter atlas developed to cluster maximally similar voxels [26]. The BOLD signal time courses were extracted from these 268 ROIs. To remove several sources of spurious variance, we used linear regression with 36 regression parameters [45] such as six motion parameters, average signals over the whole brain, white matter, and cerebrospinal fluid. Derivatives and quadratic terms were also included for all parameters. A temporal band-pass filter was applied to the time series using a first-order Butterworth filter with a pass band between 0.01 Hz and 0.08 Hz to restrict the analysis to low-frequency fluctuations, which are characteristic of rs-fMRI BOLD activity [45]. Furthermore, to reduce spurious changes in functional connectivity because of head motion, we calculated framewise displacement (FD) and removed volumes with FD > 0.5 mm, as proposed in a previous study [46]. The FD represents head motion between two consecutive volumes as a scalar quantity (the summation of absolute displacements in translation and rotation). Using the aforementioned threshold, 5.4% ± 10.6% volumes (mean [approximately 13 volumes] ± 1 SD) were removed per 10 min of rs-fMRI scanning (240 volumes) in the traveling-subject dataset; 6.2% ± 13.2 volumes were removed per rs-fMRI session in the SRPBS multidisorder dataset. If the number of volumes removed after scrubbing exceeded the average of –3 SD across participants in each dataset, the participants or sessions were excluded from the analysis. As a result, 14 sessions were removed from the traveling-subject dataset, and 20 participants were removed from the SRPBS multidisorder dataset. Furthermore, we excluded participants for whom we could not calculate functional connectivity at all 35,778 connections, primarily because of the lack of BOLD signals within an ROI. As a result, 99 participants were further removed from the SRPBS multidisorder dataset.

### Estimation of biases and factors

The participant factor (***p***), measurement bias (***m***), sampling biases (***s***_***hc***_, ***s***_***mdd***_, ***s***_***scz***_), and psychiatric disorder factor (***d***) were estimated by fitting the regression model to the functional connectivity values of all participants from the SRPBS multidisorder dataset and the traveling-subject dataset. In this instance, vectors are denoted by lowercase bold letters (e.g., ***m***), and all vectors are assumed to be column vectors. Components of vectors are denoted by subscripts such as *m*_*k*_. To represent participant characteristics, we used a 1-of-K binary coding scheme in which the target vector (e.g., **x**_***m***_) for a measurement bias ***m*** belonging to site *k* is a binary vector with all elements equal to zero—except for element *k*, which equals 1. If a participant does not belong to any class, the target vector is a vector with all elements equal to zero. A superscript T denotes the transposition of a matrix or vector, such that **x**^T^ represents a row vector. For each connectivity, the regression model can be written as follows:

$$Connectivity={x_{m}}^{T}m+{x_{s_{hc}}}^{T}s_{hc}+{x_{s_{mdd}}}^{T}s_{mdd}+{x_{s_{scz}}}^{T}s_{scz}+{x_{d}}^{T}d+{x_{p}}^{T}p+const+e,$$

$$such\;that\sum_{j}^{9}p_{j}=0,\sum_{k}^{12}m_{k}=0,\sum_{k}^{6}{s_{hc}}_{k}=0,\sum_{k}^{3}{s_{mdd}}_{k}=0,\sum_{k}^{3}{s_{scz}}_{k}=0,d_{1}(HC)=0,$$

in which ***m*** represents the measurement bias (12 sites × 1), ***s***_***hc***_ represents the sampling bias of HCs (6 sites × 1), ***s***_***mdd***_ represents the sampling bias of patients with MDD (3 sites × 1), ***s***_***scz***_ represents the sampling bias of patients with SCZ (3 sites × 1), ***d*** represents the disorder factor (3 × 1), ***p*** represents the participant factor (9 traveling subjects × 1), *const* represents the average functional connectivity value across all participants from all sites, and $e∼N(0,\gamma^{-1})$ represents noise. For each functional connectivity value, we estimated the respective parameters using regular ordinary least squares regression with L2 regularization, as the design matrix of the regression model is rank-deficient (see S3 Text). We used the “quadprog” function in MATLAB (R2016b) for estimation. When regularization was not applied, we observed spurious anticorrelation between the measurement bias and the sampling bias for HCs and spurious correlation between the sampling bias for HCs and the sampling bias for patients with psychiatric disorders (S3A Fig, left). These spurious correlations were also observed in the permutation data in which there were no associations between the site label and data (S3A Fig, right). This finding suggests that the spurious correlations were caused by the rank-deficient property of the design matrix. We tuned the hyperparameter lambda to minimize the absolute mean of these spurious correlations (S3C Fig, left).

### Analysis of contribution size

To quantitatively verify the magnitude relationship among factors, we calculated and compared the contribution size to determine the extent to which each bias type and factor explains the variance of the data in our linear model (Fig 2C). After fitting the model, the *b*-th connectivity from subject *a* can be written as follows:

$${Connectivity}_{a,b}={x_{m}^{a}}^{T}m^{b}+{x_{s_{hc}}^{a}}^{T}s_{hc}^{b}+{x_{s_{mdd}}^{a}}^{T}s_{mdd}^{b}+{x_{s_{scz}}^{a}}^{T}s_{scz}^{b}+{x_{d}^{a}}^{T}d^{b}+{x_{p}^{a}}^{T}p^{b}+const+e.$$

For example, the contribution size of measurement bias (i.e., the first term) in this model was calculated as

$${Contribution\;size}_{m}=\frac{1}{N_{m}}\frac{1}{N_{s}*N}\sum_{a=1}^{N_{s}}\sum_{b=1}^{N}\frac{{\left({x_{m}^{a}}^{T}m^{b}\right)}^{2}}{{\left({x_{m}^{a}}^{T}m^{b}\right)}^{2}+{\left({x_{s_{hc}}^{a}}^{T}s_{hc}^{b}\right)}^{2}+{\left({x_{s_{mdd}}^{a}}^{T}s_{mdd}^{b}\right)}^{2}+{\left({x_{s_{scz}}^{a}}^{T}s_{scz}^{b}\right)}^{2}+{\left({x_{d}^{a}}^{T}d^{b}\right)}^{2}+{\left({x_{p}^{a}}^{T}p^{b}\right)}^{2}+e^{2}},$$

in which *N*_*m*_ represents the number of components for each factor, *N* represents the number of connectivities, *N*_*s*_ represents the number of subjects, and *Contribution size*_*m*_ represents the magnitude of the contribution size of measurement bias. These formulas were used to assess the contribution sizes of individual factors related to measurement bias (e.g., phase-encoding direction, scanner, coil, and fMRI manufacturer: Fig 4B). We decomposed the measurement bias into these factors, after which the relevant parameters were estimated. Other parameters were fixed at the same values as previously estimated.

### Spatial characteristics of measurement bias, sampling bias, and each factor in the brain

To evaluate the spatial characteristics of each type of bias and each factor in the brain, we calculated the magnitude of the effect on each ROI. First, we calculated the median absolute value of the effect on each functional connection among sites or participants for each bias and participant factor. We then calculated the absolute value of each connection for each disorder factor. The uppercase bold letters (e.g., ***M***) and subscript vectors (e.g., ***m***_*k*_) represent the vectors for the number of functional connections:

$$M={\mathrm{median}}_{k}\left(\left|m_{k}\right|\right),S_{hc}={\mathrm{median}}_{k}\left(\left|{s_{hc}}_{k}\right|\right),S_{mdd}={\mathrm{median}}_{k}\left(\left|{s_{mdd}}_{k}\right|\right),S_{scz}={\mathrm{median}}_{k}\left(\left|{s_{scz}}_{k}\right|\right),D_{2}=|d_{2}|,D_{3}=|d_{3}|,P={\mathrm{median}}_{j}\left(\left|p_{j}\right|\right).$$

We next calculated the magnitude of the effect on ROIs as the average connectivity value between all ROIs, except for themselves.

$${Effect\_on\_ROI}_{n}=\frac{1}{N_{ROI}-1}\sum_{v\neq n}^{N_{ROI}}{Effect\_on\_connectivity}_{n,v},$$

in which *N*_*ROI*_ represents the number of ROIs, *Effect*_*on*_*ROI*_*n*_ represents the magnitude of the effect on the *n*-th ROI, and *Effect*_*on*_*connectivity*_*n*,*v*_ represents the magnitude of the effect on connectivity between the *n*-th ROI and *v*-th ROI.

### Hierarchical clustering analysis for measurement bias

We calculated the Pearson’s correlation coefficients between measurement biases ***m***_*k*_ (*N* × 1), where *N* is the number of functional connections) for each site *k*, and performed a hierarchical clustering analysis based on the correlation coefficients across measurement biases. To visualize the dendrogram (Fig 4A), we used the “dendrogram,” “linkage,” and “optimalleaforder” functions in MATLAB (R2016b).

### Comparison of models for sampling bias

We investigated whether sampling bias is caused by the differences in the number of participants among imaging sites or by sampling from different populations among imaging sites. We constructed two models and investigated which model provides the best explanation of sampling bias. In the single-population model, we assumed that participants were sampled from a single population across imaging sites. In the different-population model, we assumed that participants were sampled from different populations among imaging sites. We first theorized how the number of participants at each site affects the variance of sampling biases across connectivity values as follows:

In the single-population model, we assumed that the functional connectivity values of each participant were generated from an independent Gaussian distribution, with a mean of 0 and a variance of *ξ*^2^ for each connectivity value. Then, the functional connectivity vector for participant *j* at site *k* can be described as

$$c_{j}^{k}∼N(0,\xi^{2}I).$$

Let ***c***_*k*_ be the vector of functional connectivity at site *k* averaged across participants. In this model, ***c***_*k*_ represents the sampling bias and can be described as

$$c_{k}=\frac{1}{N_{k}}\sum_{j=1}^{N_{k}}c_{j}^{k}∼N(0,\frac{\xi^{2}}{N_{k}}I),$$

in which *N*_*k*_ represents the number of participants at site *k*. The variance across functional connectivity values for ***c***_*k*_ is described as

$$V_{k}=\frac{1}{N}\sum_{i=1}^{N}{\left(c_{ki}-\bar{c_{k}}\right)}^{2}=\frac{1}{N}{c_{k}}^{T}{\left(I-\frac{1}{N}11^{\prime }\right)}^{T}(I-\frac{1}{N}11^{\prime })c_{k}\approx \frac{1}{N}{c_{k}}^{T}c_{k},$$

in which **1** represents the *N* × 1 vector of ones and **I** represents the *N* × *N* identity matrix. Since *N* equals 35,778 and $\frac{1}{35,778}$ is sufficiently smaller than 1, we can approximate

$$I-\frac{1}{N}11^{\prime }\approx I.$$

Then, the expected value of variance across functional connectivity values for sampling bias can be described as

$$E[V_{k}]\approx \frac{1}{N}E[{c_{k}}^{T}c_{k}]=\frac{1}{N}Tr\left(\frac{\xi^{2}}{N_{k}}I\right)=\frac{\xi^{2}}{N_{k}}.$$

In the different-population model, we assumed that the functional connectivity values of each participant were generated from a different independent Gaussian distribution, with an average of ***β***_***k***_ and a variance of *ξ*^2^ depending on the population of each site. In this situation, the functional connectivity vector for participant *j* at site *k* can be described as

$$c_{j}^{k}∼N(\beta_{k},\xi^{2}I).$$

Here, we assume that the average of the population ***β***_***k***_ is sampled from an independent Gaussian distribution with an average of 0 and a variance of *σ*^2^. That is, ***β***_***k***_ is expressed as

$$\beta_{k}∼N(0,\sigma^{2}I).$$

The vector of functional connectivity for site *k* averaged across participants can then be described as

$$c_{k}∼N\left(0,(\frac{\xi^{2}}{N_{k}}+\sigma^{2})I\right).$$

The variance across functional connectivity values for ***c***_*k*_ can be described as

$$E[V_{k}]\approx \frac{\xi^{2}}{N_{k}}+\sigma^{2}.$$

In summary, the variance of sampling bias across functional connectivity values in each model is expressed by the number of participants at a given site as follows:

$$\mathrm{single}‐\mathrm{population}\;\mathrm{model}:\;y_{k}=-x_{k}+2\;\log_{10}\xi$$

$$\mathrm{different}‐\mathrm{population}\;\mathrm{model}:y_{k}=-\log_{10}\left(\xi^{2}10^{-x_{k}}+\sigma^{2}\right),$$

in which *y*_*k*_ = log_10_(*v*_*k*_), *v*_*k*_ represents the variance across functional connectivity values for ${s_{hc}}_{k}$, ${s_{hc}}_{k}$ represents the sampling bias of HCs at site *k* (*N* × 1: *N* is the number of functional connectivity), *x*_*k*_ = log_10_(*N*_*k*_), and *N*_*k*_ represents the number of participants at site *k*. We estimated the parameters *ξ* and *σ* using the MATLAB optimization function “fminunc.” To simplify statistical analyses, sampling bias was estimated based on functional connectivity in which the average across all participants was set to zero.

We aimed to determine which model provided the best explanation of sampling bias in our data by calculating the AICc (under the assumption of a Gaussian distribution) for small-sample data [36, 37], as well as BIC:

$$AICc=\sum_{k=1}^{6}\ln{\varphi_{k}}^{2}+2q+\frac{2q(q+1)}{\left(6-q-1\right)},$$

$$BIC=\sum_{k=1}^{6}\ln{\varphi_{k}}^{2}+q*log(6),$$

in which $\varphi_{k}=v_{k}-\hat{v_{k}},\;\hat{v_{k}}$ represents the estimated variance, and *q* represents the number of parameters in each model (1 or 2).

To investigate prediction performance, we used leave-one-site-out cross-validation in which we estimated the parameters *ξ* and *σ* using data from five sites. The variance of sampling bias was predicted based on the number of participants at the remaining site. This procedure was repeated to predict variance values for sampling bias at all six sites. We then calculated the absolute errors between predicted and actual variances for all sites.

### Harmonization procedures

We compared four different harmonization methods for the removal of site differences, as described in the main text.

#### Traveling-subject harmonization

Measurement biases were estimated by fitting the regression model to the combined SRPBS multidisorder and traveling-subject datasets in the same way in the “Estimation of biases and factors” section. For each connectivity, the regression model can be written as follows:

$$Connectivity={x_{m}}^{T}m+{x_{s_{hc}}}^{T}s_{hc}+{x_{s_{mdd}}}^{T}s_{mdd}+{x_{s_{scz}}}^{T}s_{scz}+{x_{d}}^{T}d+{x_{p}}^{T}p+const+e.$$ (1)

Measurement biases were removed by subtracting the estimated measurement biases. Thus, the harmonized functional connectivity values were set as follows:

$${Connectivity}^{Traveling-subject}=Connectivity-{x_{m}}^{T}\hat{m},$$

in which $\hat{m}$ represents the estimated measurement bias.

#### GLM harmonization

The GLM harmonization method adjusts the functional connectivity value for site difference using GLM. Site differences were estimated by fitting the regression model, which included site label only, to the SRPBS multidisorder dataset only. The regression model can be written as

$$Connectivity=const+{x_{s}}^{T}s^{GLM}+e,$$ (2)

in which ***s***^*GLM*^ represents the site difference (9 sites × 1). For each functional connectivity value, we estimated the parameters using regular ordinary least squares regression. Site differences were removed by subtracting the estimated site differences. Thus, the harmonized functional connectivity values were set as follows:

$${Connectivity}^{GLM}=Connectivity-{x_{s}}^{T}\hat{s^{GLM}},$$

in which $\hat{s^{GLM}}$ represents the estimated site difference.

#### Adjusted GLM harmonization

Site differences were estimated by fitting the regression model, which included site label and diagnosis label, to the SRPBS multidisorder dataset. The regression model can be written as

$$Connectivity=const+{x_{s}}^{T}s^{Adj}+{x_{d}}^{T}d^{Adj}+e,$$ (3)

In which ***s***^*Adj*^ represents the site difference (9 sites × 1). For each functional connectivity value, we estimated the parameters via regular ordinary least squares regression. Site differences were removed by subtracting the estimated site difference only. Thus, the harmonized functional connectivity values were set as follows:

$${Connectivity}^{Adj}=Connectivity-{x_{s}}^{T}\hat{s^{Adj}},$$

in which $\hat{s^{Adj}}$ represents the estimated site difference.

#### ComBat harmonization

The ComBat harmonization model [16, 17, 19, 38] extends the adjusted GLM harmonization method in two ways: (1) it models site-specific scaling factors, and (2) it uses empirical Bayesian criteria to improve the estimation of site parameters for small sample sizes. The model assumes that the expected connectivity value can be modeled as a linear combination of the biological variables and the site differences in which the error term is modulated by additional site-specific scaling factors.

$$Connectivity=const+{x_{s}}^{T}s^{ComBat}+{x_{d}}^{T}d^{ComBat}+\delta_{k}e,$$ (4)

in which ***s***^*ComBat*^ represents the site difference (9 sites × 1), and *δ*_*k*_ represents the scale parameter for site differences at site *k* for the respective connectivity value. The harmonized functional connectivity values were set as follows:

$${Connectivity}^{ComBat}=\frac{Connectivity-const-{x_{s}}^{T}\hat{s^{ComBat}}-{x_{d}}^{T}\hat{d^{ComBat}}}{\hat{\delta_{k}}}+const+{x_{d}}^{T}\hat{d^{ComBat}},$$

in which $\hat{\delta_{k}},\;\hat{d^{ComBat}}$, and $\hat{s^{ComBat}}$ are the empirical Bayes estimates of *δ*_*k*_, ***d***^*ComBat*^, and ***s***^*ComBat*^, respectively, using the “combat” function in https://github.com/Jfortin1/ComBatHarmonization. Thus, ComBat simultaneously models and estimates biological and nonbiological terms and algebraically removes the estimated additive and multiplicative site differences. Of note, in the ComBat model, we included diagnosis as covariates to preserve important biological trends in the data and avoid overcorrection.

### PCA

We developed bivariate scatterplots of the first two PCs based on a PCA of functional connectivity values in the SRPBS multidisorder dataset (Fig 6A). All participant data in the SRPBS multidisorder dataset were plotted on two axes consisting of the first two PCs (Fig 6A, small, light-colored symbols). The first two PCs could explain approximately 6% of the variance in the whole data (Fig 6B, 3.5% and 2.5% for the first and second PC, respectively). Dark-colored markers indicated the averages of the projected data across HCs at each site and the average within each psychiatric disorder in the subspace spanned by the two components. To visualize whether most of the variation in the SRPBS multidisorder dataset was still associated with imaging site after harmonization, we performed a PCA of functional connectivity values in the harmonized SRPBS multidisorder dataset (Fig 6C). We used the traveling-subject method for harmonization, as described in the following section. Finally, to visualize the measurement bias in the SRPBS multidisorder dataset, we performed a PCA of functional connectivity values in the SRPBS multidisorder data after subtracting only the sampling bias (Fig 6D).

### Twofold cross-validation evaluation procedure

We compared four different harmonization methods for the removal of site difference or measurement bias by 2-fold cross-validation, as described in the main text. In the traveling-subject harmonization method, we estimated the measurement bias by applying the regression model written in Eq 1 in the “Harmonization procedures” section to the estimating dataset. Thus, the harmonized functional connectivity values in testing dataset were set as follows:

$${connectivity}_{testing\;dataset}^{Traveling-subject}={Connectivity}_{testing\;dataset}-{x_{m}}^{T}{\hat{m}}_{estimating\;dataset},$$

in which ${\hat{m}}_{estimating\;dataset}$ represents the estimated measurement bias using the estimating dataset.

By contrast, in the other harmonization methods, we estimated the site differences by applying the regression models written in Eqs 2–4 in the “Harmonization procedures” section to the estimating dataset (fold1 data). Thus, the harmonized functional connectivity values in testing dataset were set as follows:

$${connectivity}_{testing\;dataset}^{GLM}={Connectivity}_{testing\;dataset}-{x_{s}}^{T}{\hat{s^{GLM}}}_{fold1},$$

$${connectivity}_{testing\;dataset}^{Adj}={Connectivity}_{testing\;dataset}-{x_{s}}^{T}{\hat{s^{Adj}}}_{fold1},$$

$${connectivity}_{testing\;dataset}^{ComBat}={Connectivity}_{testing\;dataset}-{x_{s}}^{T}{\hat{s^{ComBat}}}_{fold1},$$

in which ${\hat{s^{GLM}}}_{fold1},{\hat{s^{Adj}}}_{fold1},{\hat{s^{ComBat}}}_{fold1}$ represents the estimated site differences using fold1 data.

We then estimated the measurement bias, participant factor, and disorder factors by applying the regression model written in Eq 1 to the harmonized functional connectivity values in the testing dataset. Finally, we evaluated the standard deviation of the magnitude distribution of measurement bias calculated in the same way as described in the “Quantification of site differences” section among the harmonization methods. This procedure was done again by exchanging the estimating dataset and the testing dataset.

## Supporting information

S1 Data Excel spreadsheet containing, in separate sheets, the underlying numerical data for Figure panels 2A and C, 3, 4B, 5C–E, 6A–D, and 7A–C and all supporting information figures. (XLSX)

S2 Data Limitation on availability of connectivity matrices and fMRI images obtained at each site. fMRI, functional magnetic resonance imaging.
(XLSX)

S1 Text Magnitude distribution of both biases and each factor on functional connectivity. (DOCX)

S2 Text Field map correction. (DOCX)

S3 Text Selection of the regularization hyperparameter lambda. (DOCX)

S4 Text Brain regions contributing the measurement bias of each site. (DOCX)

S5 Text Classifiers for MDD and SCZ, based on the four harmonization methods. MDD, major depressive disorder; SCZ, schizophrenia.
(DOCX)

S6 Text Regression models of participant age based on the four harmonization methods. (DOCX)

S1 Fig Distributions and statistics for each type of bias and each factor. (A, B) The distribution of the effects of each bias and each factor on functional connectivity vectors. Functional connectivity was measured based on Fisher’s z-transformed Pearson’s correlation coefficients. The *x* axis represents the effect size of the Fisher’s z-transformed Pearson’s correlation coefficients. In (A) and (B), the *y* axis represents the density of connectivity and the log-transformed the number of connections, respectively. Each line represents one participant or one site. ASD, autism spectrum disorder; HC, healthy controls; MDD, major depressive disorder; SCZ, schizophrenia.
(TIF)

S2 Fig Effects of field map correction. (A) Top: Mean effects of connectivity at all 268 ROIs with field map correction. Color-coding follows that for Fig 4 in the main text. Difference between field map–corrected and field map–uncorrected datasets for participant factor (middle) and measurement bias (bottom). Red represents positive effects due to correction (i.e., increase in participant factor and decrease in measurement bias). Blue represents negative effects (i.e., decrease in participant factor and increase in measurement bias). (B) The standard deviations of participant factor and measurement bias after field map correction. Bars represent the average, whereas error bars represent the standard deviation across sites or participants. Each data point represents one participant or one site. (C) Clustering dendrogram for measurement bias after field map correction. The height of each linkage in the dendrogram represents the distance between the clusters joined by that link. ATT, Siemens TimTrio scanner at Advanced Telecommunications Research Institute International; ATV, Siemens Verio scanner at Advanced Telecommunications Research Institute International; COI, Center of Innovation in Hiroshima University; KPM, Kyoto Prefectural University of Medicine; KUS, Siemens Skyra scanner at Kyoto University; KUT, Siemens TimTrio scanner at Kyoto University; ROI, region of interest; SWA, Showa University; UTO, University of Tokyo; YC1, Yaesu Clinic 1.
(TIF)

S3 Fig Selection of the regularization hyperparameter lambda. (A) Correlation matrix between measurement biases and sampling biases in HCs and matrices between sampling biases of HCs and sampling biases of patients with psychiatric disorders at lambda = 0 and lambda = 14, 12 (left: real data, right: permutation data). (B) Correlation values between the two types of bias as functions of lambda from 0 to 20 (left: real data, right: permutation data). Correlations were calculated between the measurement and sampling biases of HCs and between the sampling biases of HCs and sampling biases of patients with psychiatric disorders. (C) Absolute mean of three correlations as a function of lambda. (D) Percentage change in the residual error between model and real data as a function of lambda. ATT, Siemens TimTrio scanner at Advanced Telecommunications Research Institute International; ATV, Siemens Verio scanner at Advanced Telecommunications Research Institute International; COI, Center of Innovation in Hiroshima University; KUT, Siemens TimTrio scanner at Kyoto University; MDD, major depressive disorder; SCZ, schizophrenia; SWA, Showa University; UTO, University of Tokyo1.
(TIF)

S4 Fig Spatial distribution of the measurement bias of each site in various brain regions. Mean effects of connectivity for all 268 ROIs. For each ROI, the mean effects of all functional connections associated with that ROI were calculated for the measurement bias of each site. Warmer (red) and cooler (blue) colors correspond to large and small effects, respectively. The magnitudes of the effects are normalized within each site (*z*-score). ATT, Siemens TimTrio scanner at Advanced Telecommunications Research Institute International; ATV, Siemens Verio scanner at Advanced Telecommunications Research Institute International; COI, Center of Innovation in Hiroshima University; HKH, Hiroshima Kajikawa Hospital; HUH: Hiroshima University Hospital; KPM, Kyoto Prefectural University of Medicine; KUS, Siemens Skyra scanner at Kyoto University; KUT, Siemens TimTrio scanner at Kyoto University; ROI, region of interest; SWA, Showa University; UTO, University of Tokyo; YC1, Yaesu Clinic 1; YC2, Yaesu Clinic 2.
(TIF)

S5 Fig Performance of disorder classifiers and age regression model in the training dataset. (A, B) Performance of each classifier in the training dataset for each harmonization method (blue for MDD, red for SCZ). Bars represent the average, whereas error bars represent the standard deviation across 100 resamplings. (C) Scatterplot of actual age and predicted age for each harmonization method. The solid line represents the linear regression of the actual age from the predicted age. The MAE and correlation coefficient (*r*) are also shown. Each data point represents one participant. AUC, area under the curve; MAE, mean absolute error; MCC, Matthews correlation coefficient; MDD, major depressive disorder; SCZ, schizophrenia.
(TIF)

S6 Fig Classifier performances for MDD and SCZ for different harmonization methods. (A) The probability distribution for the diagnosis of MDD in the training dataset (left) and independent cohort (right) for each harmonization method. The MDD and HC distributions are depicted in blue and gray, respectively. (B) The probability distribution for the diagnosis of SCZ in the training dataset (left) and independent cohort (right) for each harmonization method. The SCZ and HC distributions are depicted in red and gray, respectively. (C, D) Classifier performance in the independent cohort for each harmonization method and each classifier (blue for MDD, red for SCZ). AUC, area under the curve; HC, healthy control; MCC, Matthews correlation coefficient; MDD, major depressive disorder; SCZ, schizophrenia.
(TIF)

S7 Fig Performance of a regression model for the prediction of a participant’s age for different harmonization methods. Scatterplots of actual age and predicted age. The solid line indicates the linear regression of the actual age from the predicted age. The MAE and correlation coefficient (*r*) are shown in each panel. Each data point represents one participant. Each panel shows the results for the (A) traveling-subject method, (B) ComBat method, (C) GLM method, (D) adjusted GLM method, or (E) raw method (i.e., the data were not harmonized across sites). GLM, general linear model; MAE, mean absolute error.
(TIF)

S1 Table Imaging protocols for resting-state fMRI in the traveling-subject dataset. fMRI, functional magnetic resonance imaging.
(XLSX)

S2 Table Demographic characteristics of patients in the independent validation cohort dataset for MDD prediction. MDD, major depressive disorder.
(XLSX)

S3 Table Demographic characteristics of patients in the independent validation cohort dataset for SCZ prediction. SCZ, schizophrenia.
(XLSX)

S4 Table Demographic characteristics of patients in the independent validation cohort dataset for age prediction. (XLSX)

S5 Table Imaging protocols for resting-state fMRI in the independent validation cohort dataset. fMRI, functional magnetic resonance imaging.
(XLSX)

S6 Table Comparison between the variances of distributions in measurement bias and disorder factor. (XLSX)

S7 Table Comparison between variances of the distributions in sampling bias for healthy control and disorder factors. (XLSX)

S8 Table Results of MDD prediction. MDD, major depressive disorder.
(XLSX)

S9 Table All results for MDD prediction at each site. MDD, major depressive disorder.
(XLSX)

S10 Table Results of SCZ prediction. SCZ, schizophrenia.
(XLSX)

S11 Table All results for SCZ prediction at each site. SCZ, schizophrenia.
(XLSX)

## Abbreviations

A→P: anterior to posterior
ABIDE: Autism Brain Imaging Data Exchange
AICc: corrected Akaike information criterion
AMED: Japan Agency for Medical Research and Development
ASD: autism spectrum disorder
BIC: Bayesian information criterion
BOLD: blood-oxygen-level dependent
CRHD: Connectomes Related to Human Disease
DecNef: Decoded Neurofeedback
GE: GE functional magnetic resonance imaging
GLM: generalized linear model
HC: healthy control
HCP: Human Connectome Project
HUH: Hiroshima University Hospital
MDD: major depressive disorder
OCD: obsessive-compulsive disorder
P→A: posterior to anterior
PC: principal component
PCA: principal component analysis
PHI: Philips functional magnetic resonance imaging
ROI: region of interest
rs-fcMRI: resting-state functional connectivity MRI
rs-fMRI: resting-state functional magnetic resonance imaging
SCZ: schizophrenia
SIE: Siemens functional magnetic resonance imaging
SRPBS: Strategic Research Program for Brain Sciences
SWA: Showa University TE, echo time
TR: repetition time
