# Supplementary material for: Harmonization of resting-state functional MRI data across multiple imaging sites via the separation of site differences into sampling bias and measurement bias
Source: PLoS Biol. 2019 Apr 18;17(4):e3000042. doi: 10.1371/journal.pbio.3000042 (PMC6472734; doi:10.1371/journal.pbio.3000042)
Supplement: S1 Text — (DOCX) [file pbio.3000042.s003.docx]

**S1 Text. Magnitude distribution of both biases and each factor on functional connectivity.**

To quantitatively evaluate the effect of measurement and sampling biases on functional connectivity, we compared the magnitudes of both types of bias with the magnitudes of psychiatric disorders and participant factors. For this purpose, we investigated the magnitude distribution of both biases, as well as the effects of psychiatric disorders and participant factors on functional connectivity overall 35,778 elements in a 35,778-dimensional vector to see how many functional connectivities were largely affected. S1a Fig: the *x*-axis shows the magnitude as Fisher’s *z*-transformed Pearson’s correlation coefficients, while the *y*-axis shows the density of the number of connectivities. S1b Fig shows the same data, except the *y*-axis represents the log-transformed number of connectivities for better visualization of small values. There were significant differences among biases and factors for larger magnitudes near the tails of their distributions. For example, the number of connectivities, which was largely affected (i.e., a magnitude larger than 0.2), was more than 100 for the participant factor, approximately 100 for measurement bias, and nearly 0 for all sampling biases, as well as all disorder factors.
