# Supplementary material for: Harmonization of resting-state functional MRI data across multiple imaging sites via the separation of site differences into sampling bias and measurement bias
Source: PLoS Biol. 2019 Apr 18;17(4):e3000042. doi: 10.1371/journal.pbio.3000042 (PMC6472734; doi:10.1371/journal.pbio.3000042)
Supplement: S2 Text — (DOCX) [file pbio.3000042.s004.docx]

**S2 Text. Field map correction.**

We investigated the effect of field-map correction on data harmonization [1-3]. We used SPM12 for field-map correction, in accordance with the SPM protocol. A total of 35,778 functional connections were calculated from echo-planar images (EPIs) following field-map correction. Participant factors and measurement biases were estimated by fitting the regression model to the traveling-subject dataset only. The regression model can be described as follows:

$$Connectivity=const+{\mathbf{x}_{\boldsymbol{p}}}^{T}\boldsymbol{p}+{\mathbf{x}_{\boldsymbol{m}}}^{T}\boldsymbol{m}+e,$$

$$such that \sum_{j}^{9} p_{j}=0,\sum_{k}^{12} m_{k}=0.$$

To evaluate the spatial effect of field-map correction on various brain regions, we visualized the difference in the effect on each ROI between datasets with and without field-map correction. We also visualized the effect of measurement bias on each ROI using data subjected to field-map correction (S2a Fig). We calculated the standard deviation of the measurement bias and the participant factor and compared the results between datasets with and without field-map correction (S2b Fig). Furthermore, we performed hierarchical clustering analysis of measurement bias in the dataset subjected to field-map correction (S2c Fig). S1a and S1b Figs demonstrate that field-map correction remarkably reduced the effect of measurement bias in the cerebellum and lower regions of the frontal cortex, while also increasing the effect of the participant factor. However, the presence of the cluster for phase-encoding direction in S2c Fig indicates that field-map correction did not completely eliminate the influence of the difference in the phase-encoding direction.

**References**

1. Hutton C, Bork A, Josephs O, Deichmann R, Ashburner J, Turner R. Image distortion correction in fMRI: A quantitative evaluation. Neuroimage. 2002;16(1):217-40. doi: 10.1006/nimg.2001.1054. PubMed PMID: 11969330.

2. Jenkinson M. Fast, automated, N-dimensional phase-unwrapping algorithm. Magn Reson Med. 2003;49(1):193-7. doi: 10.1002/mrm.10354. PubMed PMID: 12509838.

3. Jezzard P, Balaban RS. Correction for geometric distortion in echo planar images from B0 field variations. Magn Reson Med. 1995;34(1):65-73. Epub 1995/07/01. PubMed PMID: 7674900.
