# Supplementary material for: Harmonization of resting-state functional MRI data across multiple imaging sites via the separation of site differences into sampling bias and measurement bias
Source: PLoS Biol. 2019 Apr 18;17(4):e3000042. doi: 10.1371/journal.pbio.3000042 (PMC6472734; doi:10.1371/journal.pbio.3000042)
Supplement: S3 Text — (DOCX) [file pbio.3000042.s005.docx]

**S3 Text. Selection of the regularization hyper-parameter lambda.**

Because the design matrix of the regression model was rank-deficient, *L2* regularization was applied when estimating each type of bias and factor. When regularization was not applied, we observed spurious anti-correlation between measurement bias and sampling bias for healthy controls, as well as spurious correlation between sampling bias for healthy controls and sampling bias for patients with psychiatric disorders (S3a Fig, left). These spurious correlations can even be observed in the permutation data, in which there were no associations between site label and data (S3a Fig, right). This suggests that the spurious correlations were caused by the rank-deficient property of the design matrix. We utilized the hyper-parameter lambda to minimize the absolute mean of these spurious correlations (S3c Fig, left). We confirmed that the values of lambda for the real data were almost identical to those for the permutation data (S3c Fig, right). Furthermore, although the ability to explain the data decreases when using regularization, we confirmed that the degree of decrease due to regularization was less than 1% (S3d Fig).
