# Supplementary material for: Harmonization of resting-state functional MRI data across multiple imaging sites via the separation of site differences into sampling bias and measurement bias
Source: PLoS Biol. 2019 Apr 18;17(4):e3000042. doi: 10.1371/journal.pbio.3000042 (PMC6472734; doi:10.1371/journal.pbio.3000042)
Supplement: S4 Text — (DOCX) [file pbio.3000042.s006.docx]

**S4 Text. Brain regions contributing the measurement bias of each site.**

To evaluate the spatial distribution of the measurement bias of each site in the whole brain, we utilized the same method in Fig 3 in the main text. We projected connectivity information to anatomical regions of interest (ROIs). S4 Fig shows the relative contribution of individual ROIs to measurement bias of each site in the whole brain.
