# Supplementary material for: Harmonization of resting-state functional MRI data across multiple imaging sites via the separation of site differences into sampling bias and measurement bias
Source: PLoS Biol. 2019 Apr 18;17(4):e3000042. doi: 10.1371/journal.pbio.3000042 (PMC6472734; doi:10.1371/journal.pbio.3000042)
Supplement: S5 Text — MDD, major depressive disorder; SCZ, schizophrenia. (DOCX) [file pbio.3000042.s007.docx]

**S5 Text. Classifiers for MDD and SCZ, based on the four harmonization methods.**

To quantitatively evaluate the harmonization method, we constructed biomarkers for psychiatric disorders using the SRPBS multi-disorder dataset, which distinguishes between HCs and patients, based on resting-state functional connectivity (rs-fcMRI). We compared four different harmonization methods for the removal of site difference from the SRPBS multi-disorder dataset: (1) by using a traveling-subject method; (2) by using a ComBat method; (3) by using a GLM method; and (4) by using an Adjusted GLM. We also compared these four methods to the non-harmonization method (Raw method).

We aimed to focus on multi-site data; therefore, we targeted data from patients with MDD and SCZ who were sampled from multiple sites. To construct each classifier, a machine-learning technique was applied to (1) all functional connectivity data for HCs and patients with MDD from the SRPBS multi-disorder dataset (425 HCs from nine sites and 135 patients with MDD from five sites; Table 1 in the main text) or (2) all functional connectivity data for HCs and patients with SCZ from the SRPBS multi-disorder dataset (425 HCs from nine sites and 44 patients with SCZ from three sites; Table 1 in the main text). Based on our previous results, we assumed that disorder factors were not associated with whole-brain connectivity, but with a specific subset of connections [1]. Therefore, we conducted logistic regression analyses using the least absolute shrinkage and selection operator (LASSO) method to select the optimal subset of functional connections from among 35,778 connections. A logistic function was used to define the probability of a participant belonging to the MDD (or SCZ) class, as follows:

$P_{sub}\left( y_{sub}=1|\boldsymbol{c}_{sub};\boldsymbol{w} \right)=\frac{1}{1+\exp\left( -\boldsymbol{w}^{T}\boldsymbol{c}_{sub} \right)}$,

in which $y_{sub}$ represents the class label (MDD or SCZ, *y* = 1; HC, *y* = 0) of a participant, $\boldsymbol{c}_{sub}$ represents a functional connectivity vector for a given participant, and ***w*** represents the weight vector. The weight vector ***w*** was determined so as to minimize

$$J\left( \mathbf{w} \right)=-\frac{1}{n_{sub}}\sum_{j=1}^{n_{sub}} \log P_{j}\left( y_{j}=1|\boldsymbol{c}_{\boldsymbol{j}};\boldsymbol{w} \right)+\lambda\left\| \boldsymbol{w} \right\|_{1},$$

in which $\left\| \boldsymbol{w} \right\|_{1}=\sum_{i}^{N} \left| w_{i} \right|$ and $\lambda$ represent hyper-parameters that controls the amount of shrinkage applied to the estimates. To estimate $\lambda$ properly, we used the “*lassoglm*” function in MATLAB (R2015a, Mathworks, USA) and set “NumLambda” = 25 and “CV” = 10.

Because the SRPBS multi-disorder dataset is unbalanced with regard to the numbers of patients and HCs, we used the under-sampling [2] and resampling method to develop and evaluate classifiers. To train the MDD classifier, 130 patients with MDD and 130 HCs were randomly sampled from the dataset, and classifier performance was tested among the remaining participants. For the SCZ classifier, data were randomly sampled for 40 patients with SCZ and 40 HCs. We calculated the area under the curve (AUC), accuracy, sensitivity, and specificity. Furthermore, to properly evaluate classifier performance even for the unbalanced dataset, we calculated the Matthews correlation coefficients (MCC) [3, 4] as indicators of classifier performance. MCC correctly takes into account the ratio of the confusion matrix size. Especially in unbalanced datasets, the MCC is able to identify whether prediction is proceeding appropriately, whereas accuracy is not. Under-sampling is disadvantageous in that it does not allow the classifier to learn using the excluded data. Therefore, to ensure all participants were used during classifier training, we repeated the aforementioned procedure 100 times (i.e., resampling), and the average value of classifier performance was considered indicative of classifier performance in the training dataset. S5a and S5b Figs show the classifier performances and the left panels of S6a and S6b Figs depict the distribution of the average probability for a given disorder. The generalizability of the models was tested using parts of the dataset for the completely independent validation cohort obtained from the following sites: Hiroshima Rehabilitation Center (HRC) and Yamaguchi University (UYA) (47 HCs and 12 patients with MDD from HRC; 117 HCs and 76 patients with MDD from UYA) for the MDD classifier; Kyoto University Trio (KTT) for the SCZ classifier (61 HCs and 36 patients with SCZ from KTT) (see S2, 3 and 5 Tables). Since we created 100 classifiers using the training data, we entered the independent cohort data into all trained classifiers and averaged the resultant probability values. For each participant, when the average probability was greater than 0.5, the diagnostic class label *y* of this participant was set equal to 1 (MDD or SCZ, *y* = 1; HC, *y* = 0). Classifier performance in the independent cohort is shown in S6c and S6d Figs and the right panels of S6a and S6b Figs depict the distribution of the average probability for a given disorder. Further details of classifier performance are presented in S8–11 Tables.

Classifier output was defined as the probability of a participant being categorized into the MDD or SCZ class. Diagnostic probability values greater than 0.5 were considered indicative of a psychiatric disorder. The distribution of diagnostic probability in the training dataset (left panels in S6a and 6b Figs) revealed that patients with psychiatric disorders and HCs were clearly separated by a threshold of 0.5 (the middle line in each panel) for all methods. By contrast, the distribution of diagnostic probability in the independent cohort (right panels in S6a and 6b Figs) revealed that patients with psychiatric disorders and HCs were separated by a threshold of 0.5 only for the traveling-subject and ComBat methods. No such separation was observed for the other methods because of the leftward shift of the distributions for HCs and patients. Thus, in the independent cohort, very low sensitivity (below 0.5) and unduly high specificity were observed for the GLM and adjusted GLM methods, whereas medium sensitivity (approximately 0.5) and unduly high specificity were observed for the raw methods for the MDD and SCZ classifiers (S6c and 6d Figs). Unduly high specificity was achieved because patients and HCs were indifferently classified as HCs. This result indicates that the GLM and adjusted GLM methods are unable to remove site differences and may even negatively impact classification (see the Discussion section for more details) [5, 6].

We next compared the generalizability of the traveling-subject, ComBat, and raw methods. Classifier performance was evaluated using MCC [3, 4] because our datasets were unbalanced with regard to the number of patients and HCs. The MCC is suitable for evaluating unbalanced datasets because MCC correctly takes into account the ratio of the confusion matrix size. The traveling-subject method was superior to the raw method for MDD and SCZ classifiers. The ComBat method was also superior to the raw method for the MDD classifier but inferior to the raw method for the SCZ classifier. The index values for the MDD classifier were as follows for MCC: 0.376 (ComBat method) > 0.348 (traveling-subject method) > 0.267 (raw method). The values for the SCZ classifier were as follows for the MCC: 0.520 (traveling-subject method) > 0.474 (raw method) > 0.400 (ComBat method). In the traveling-subject and ComBat methods, the threshold of 0.5 was nearly correctly set at the approximate intersection between the HC and patient distributions, whereas the threshold was shifted slightly rightward in the raw method. These results indicate that harmonization of the SRPBS multi-disorder dataset, based on the traveling-subject and ComBat methods, outperformed other harmonization methods with regard to classifier generalizability. However, because the ComBat method was inferior to even the raw method for the SCZ classifier, ComBat may not be appropriate for certain datasets.

**References**

1. Yahata N, Morimoto J, Hashimoto R, Lisi G, Shibata K, Kawakubo Y, et al. A small number of abnormal brain connections predicts adult autism spectrum disorder. Nat Commun. 2016;7:11254. Epub 2016/04/15. doi: 10.1038/ncomms11254. PubMed PMID: 27075704; PubMed Central PMCID: PMCPMC4834637.

2. Wallace BC, Small K, Brodley CE, Trikalinos TA. Class Imbalance, Redux. 2011:754-63. doi: 10.1109/icdm.2011.33.

3. Chicco D. Ten quick tips for machine learning in computational biology. BioData Min. 2017;10:35. doi: 10.1186/s13040-017-0155-3. PubMed PMID: 29234465; PubMed Central PMCID: PMCPMC5721660.

4. Matthews BW. Comparison of the predicted and observed secondary structure of T4 phage lysozyme. Biochimica et biophysica acta. 1975;405(2):442-51. Epub 1975/10/20. PubMed PMID: 1180967.

5. Fortin JP, Cullen N, Sheline YI, Taylor WD, Aselcioglu I, Cook PA, et al. Harmonization of cortical thickness measurements across scanners and sites. Neuroimage. 2017;167:104-20. doi: 10.1016/j.neuroimage.2017.11.024. PubMed PMID: 29155184.

6. Rao A, Monteiro JM, Mourao-Miranda J, Alzheimer's Disease I. Predictive modelling using neuroimaging data in the presence of confounds. Neuroimage. 2017;150:23-49. doi: 10.1016/j.neuroimage.2017.01.066. PubMed PMID: 28143776; PubMed Central PMCID: PMCPMC5391990.
