# Supplementary material for: Harmonization of resting-state functional MRI data across multiple imaging sites via the separation of site differences into sampling bias and measurement bias
Source: PLoS Biol. 2019 Apr 18;17(4):e3000042. doi: 10.1371/journal.pbio.3000042 (PMC6472734; doi:10.1371/journal.pbio.3000042)
Supplement: S6 Text — (DOCX) [file pbio.3000042.s008.docx]

**S6 Text. Regression models of participant age based on the four harmonization methods.**

To further investigate the effectiveness of the harmonization methods, we constructed regression models to predict participant age using four different harmonization methods, as well as the raw method, and compared prediction performance among the models. To construct each regression model, a machine-learning technique was applied to all functional connectivity data from HCs (425 HCs from nine sites; Table 1 in the main text). We then employed linear regression using the LASSO method, as follows:

$$y_{sub}=\boldsymbol{w}^{T}\boldsymbol{c}_{sub},$$

in which $y_{sub}$ represents the age of the participant, $\boldsymbol{c}_{sub}$ represents a functional connectivity vector of the participant, and ***w*** represents the weight vector of the linear regression. The performance of linear regression in the training data was evaluated via 10-fold cross validation. We calculated the MAE and Pearson’s correlation coefficients between predicted age and actual age. The generalizability of the models was examined using parts of the completely independent validation cohort dataset obtained from the ATR TimTrio, ATR Verio, and ATR Prisma sites (223 HCs, S4 and 5 Tables). This dataset is collected for different purpose from this study and contains subjects from a wide range of ages (20–69). We developed 10 linear regression models using the training data (each cross validation); therefore, we entered the independent cohort data into all trained linear regression models. The average output was regarded as the predicted age. We also calculated the MAE and Pearson’s correlation coefficients between the predicted age and the actual age. S7 Fig shows the scatter plot of the actual age and the predicted age in the independent cohort. S5c Fig shows the same plot in the training dataset.

The ComBat method achieved the lowest mean absolute error (MAE) value and the highest *r* value, whereas the traveling-subject method achieved the second lowest MAE value and the second highest *r* value (S7 Fig). Furthermore, the MAE values of the ComBat and traveling-subject methods were significantly lower than that of the raw method (two-tailed paired *t*-test; ComBat: *p* = 3.1 $\times$ 10^-20^, *t* = -10.18, *df* = 222; traveling-subject: *p* = 6.3 $\times$ 10^-8^, *t* = -5.5, *df* = 222). These results indicate that the ComBat and traveling-subject methods outperformed the other harmonization methods for constructing a regression model to predict a participant’s age with regard to generalizability.
