# Supplementary figures and images for: Harmonization of resting-state functional MRI data across multiple imaging sites via the separation of site differences into sampling bias and measurement bias
Source: PLoS Biol. 2019 Apr 18;17(4):e3000042. doi: 10.1371/journal.pbio.3000042 (PMC6472734; doi:10.1371/journal.pbio.3000042)

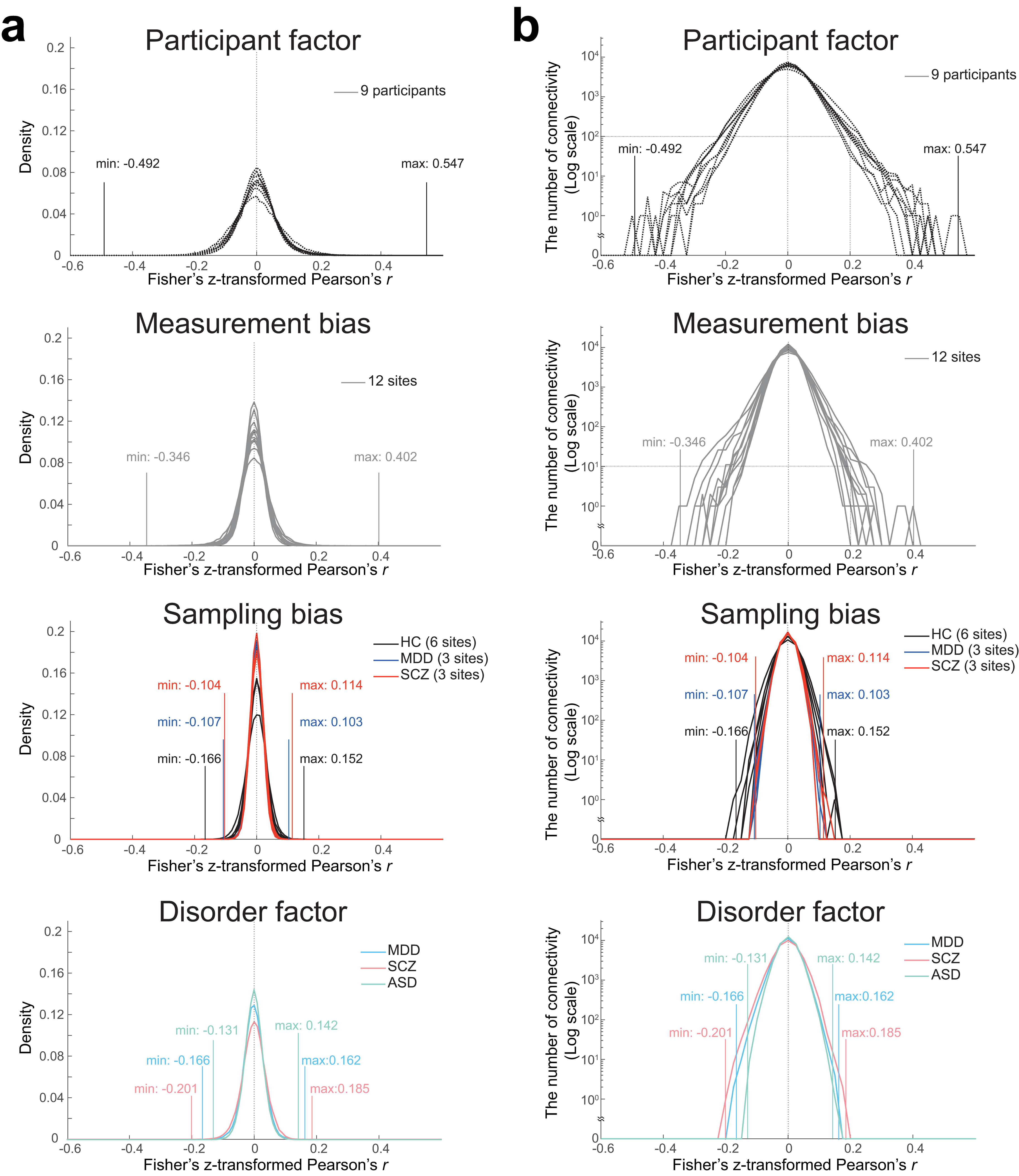

Supplement: S1 Fig — (A, B) The distribution of the effects of each bias and each factor on functional connectivity vectors. Functional connectivity was measured based on Fisher’s z-transformed Pearson’s correlation coefficients. The x axis represents the effect size of the Fisher’s z-transformed Pearson’s correlation coefficients. In (A) and (B), the y axis represents the density of connectivity and the log-transformed the number of connections, respectively. Each line represents one participant or one site. ASD, autism spectrum disorder; HC, healthy controls; MDD, major depressive disorder; SCZ, schizophrenia. (TIF) [file pbio.3000042.s009.tif]

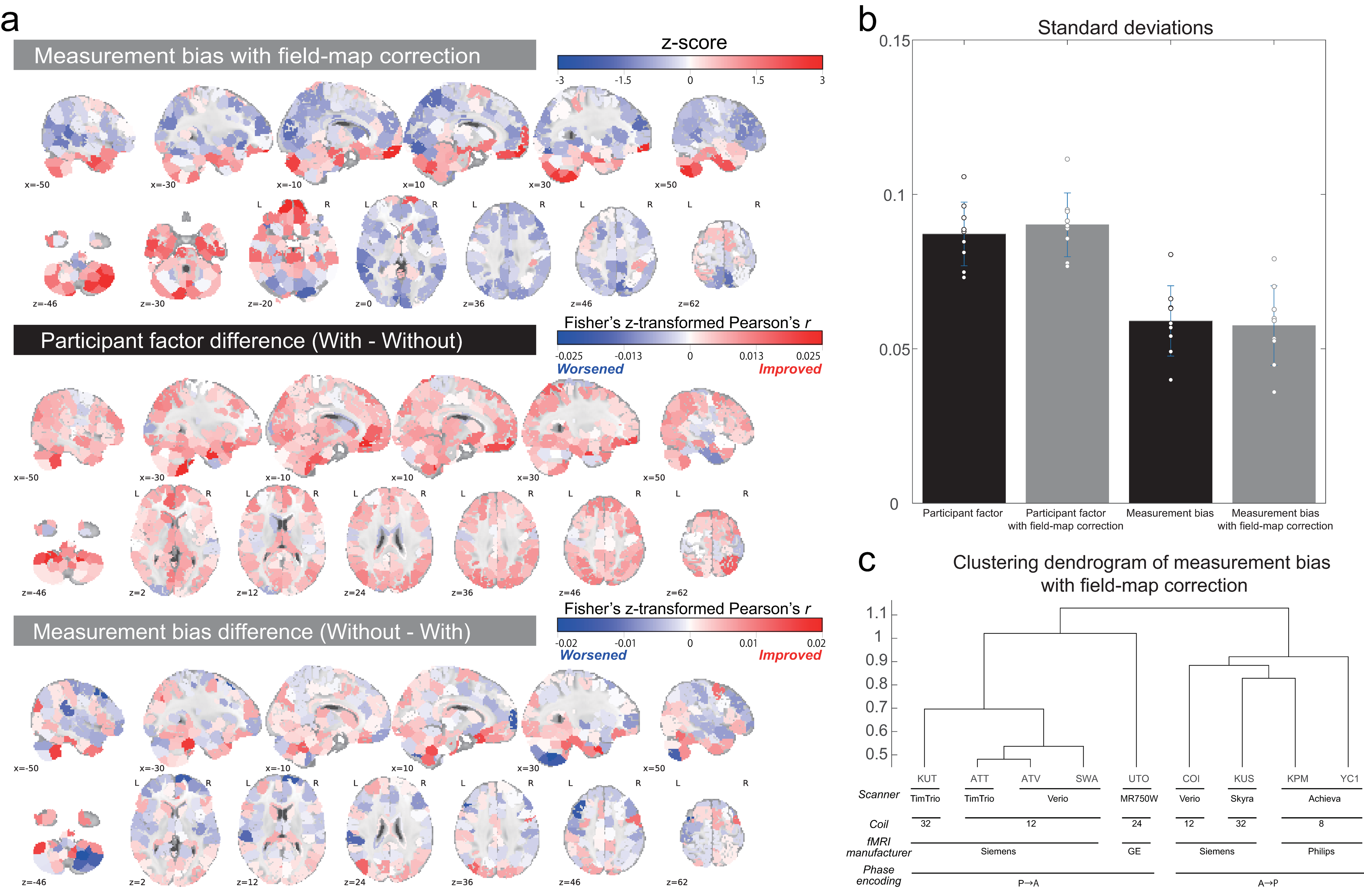

Supplement: S2 Fig — (A) Top: Mean effects of connectivity at all 268 ROIs with field map correction. Color-coding follows that for Fig 4 in the main text. Difference between field map–corrected and field map–uncorrected datasets for participant factor (middle) and measurement bias (bottom). Red represents positive effects due to correction (i.e., increase in participant factor and decrease in measurement bias). Blue represents negative effects (i.e., decrease in participant factor and increase in measurement bias). (B) The standard deviations of participant factor and measurement bias after field map correction. Bars represent the average, whereas error bars represent the standard deviation across sites or participants. Each data point represents one participant or one site. (C) Clustering dendrogram for measurement bias after field map correction. The height of each linkage in the dendrogram represents the distance between the clusters joined by that link. ATT, Siemens TimTrio scanner at Advanced Telecommunications Research Institute International; ATV, Siemens Verio scanner at Advanced Telecommunications Research Institute International; COI, Center of Innovation in Hiroshima University; KPM, Kyoto Prefectural University of Medicine; KUS, Siemens Skyra scanner at Kyoto University; KUT, Siemens TimTrio scanner at Kyoto University; ROI, region of interest; SWA, Showa University; UTO, University of Tokyo; YC1, Yaesu Clinic 1. (TIF) [file pbio.3000042.s010.tif]

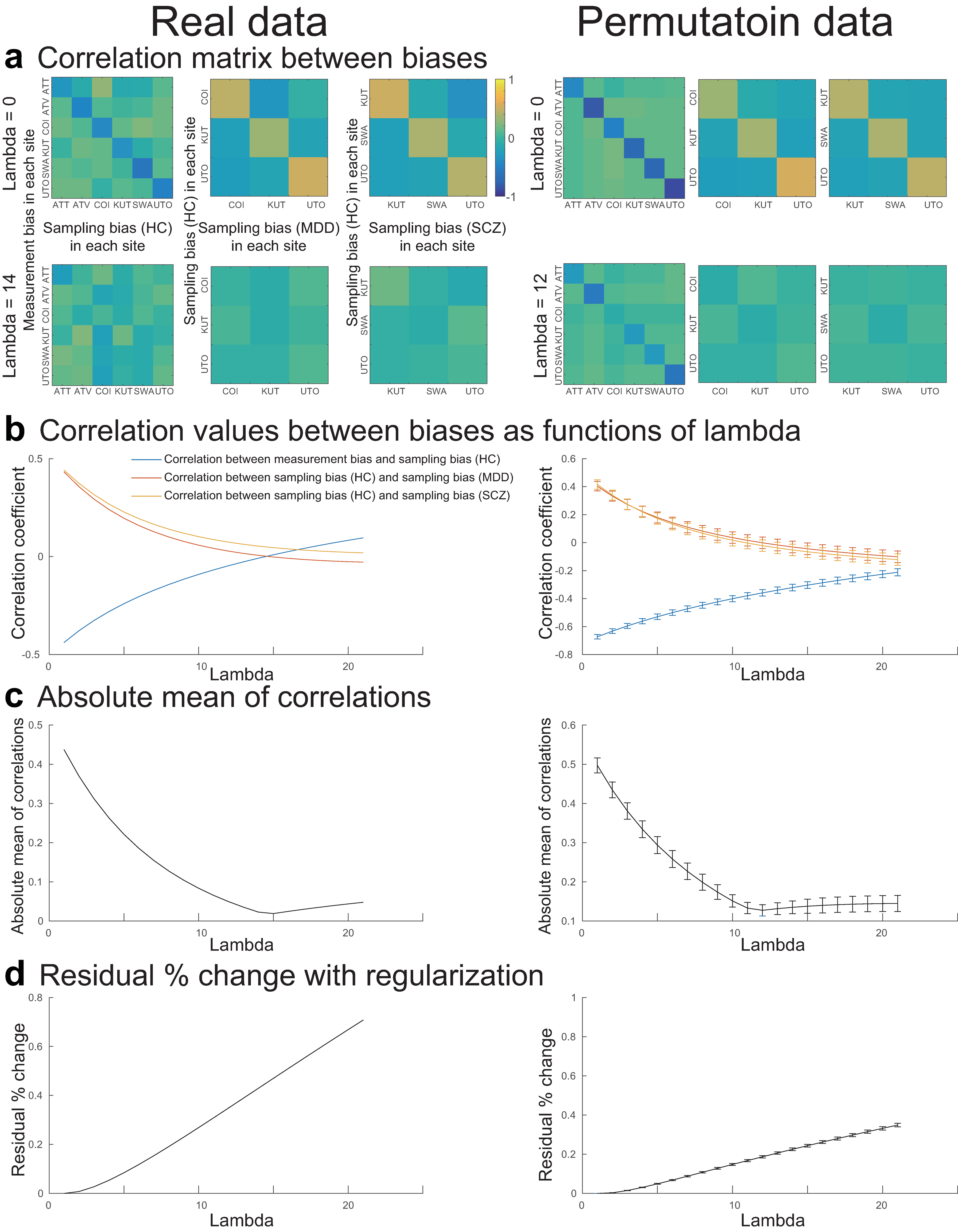

Supplement: S3 Fig — (A) Correlation matrix between measurement biases and sampling biases in HCs and matrices between sampling biases of HCs and sampling biases of patients with psychiatric disorders at lambda = 0 and lambda = 14, 12 (left: real data, right: permutation data). (B) Correlation values between the two types of bias as functions of lambda from 0 to 20 (left: real data, right: permutation data). Correlations were calculated between the measurement and sampling biases of HCs and between the sampling biases of HCs and sampling biases of patients with psychiatric disorders. (C) Absolute mean of three correlations as a function of lambda. (D) Percentage change in the residual error between model and real data as a function of lambda. ATT, Siemens TimTrio scanner at Advanced Telecommunications Research Institute International; ATV, Siemens Verio scanner at Advanced Telecommunications Research Institute International; COI, Center of Innovation in Hiroshima University; KUT, Siemens TimTrio scanner at Kyoto University; MDD, major depressive disorder; SCZ, schizophrenia; SWA, Showa University; UTO, University of Tokyo1. (TIF) [file pbio.3000042.s011.tif]

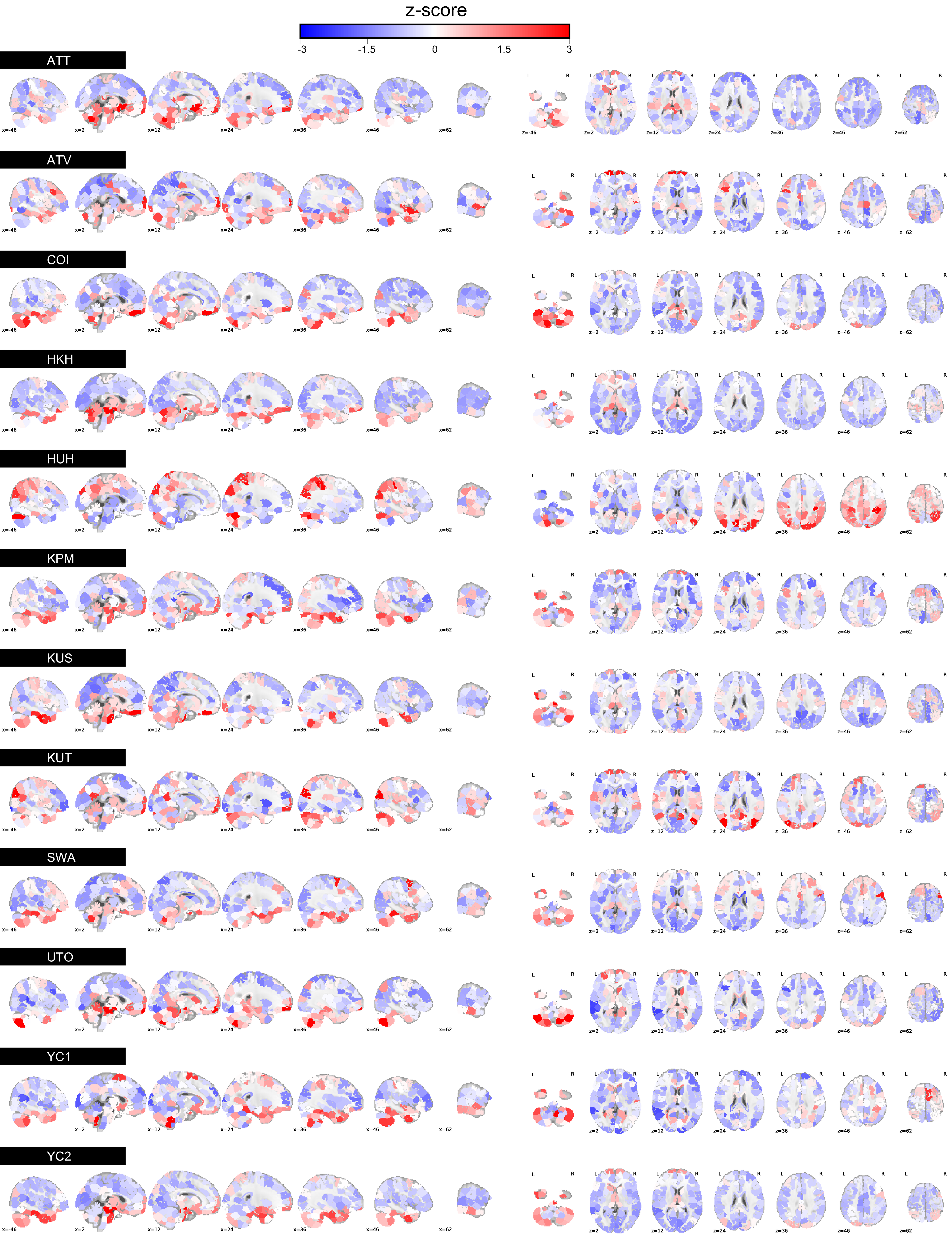

Supplement: S4 Fig — Mean effects of connectivity for all 268 ROIs. For each ROI, the mean effects of all functional connections associated with that ROI were calculated for the measurement bias of each site. Warmer (red) and cooler (blue) colors correspond to large and small effects, respectively. The magnitudes of the effects are normalized within each site (z-score). ATT, Siemens TimTrio scanner at Advanced Telecommunications Research Institute International; ATV, Siemens Verio scanner at Advanced Telecommunications Research Institute International; COI, Center of Innovation in Hiroshima University; HKH, Hiroshima Kajikawa Hospital; HUH: Hiroshima University Hospital; KPM, Kyoto Prefectural University of Medicine; KUS, Siemens Skyra scanner at Kyoto University; KUT, Siemens TimTrio scanner at Kyoto University; ROI, region of interest; SWA, Showa University; UTO, University of Tokyo; YC1, Yaesu Clinic 1; YC2, Yaesu Clinic 2. (TIF) [file pbio.3000042.s012.tif]

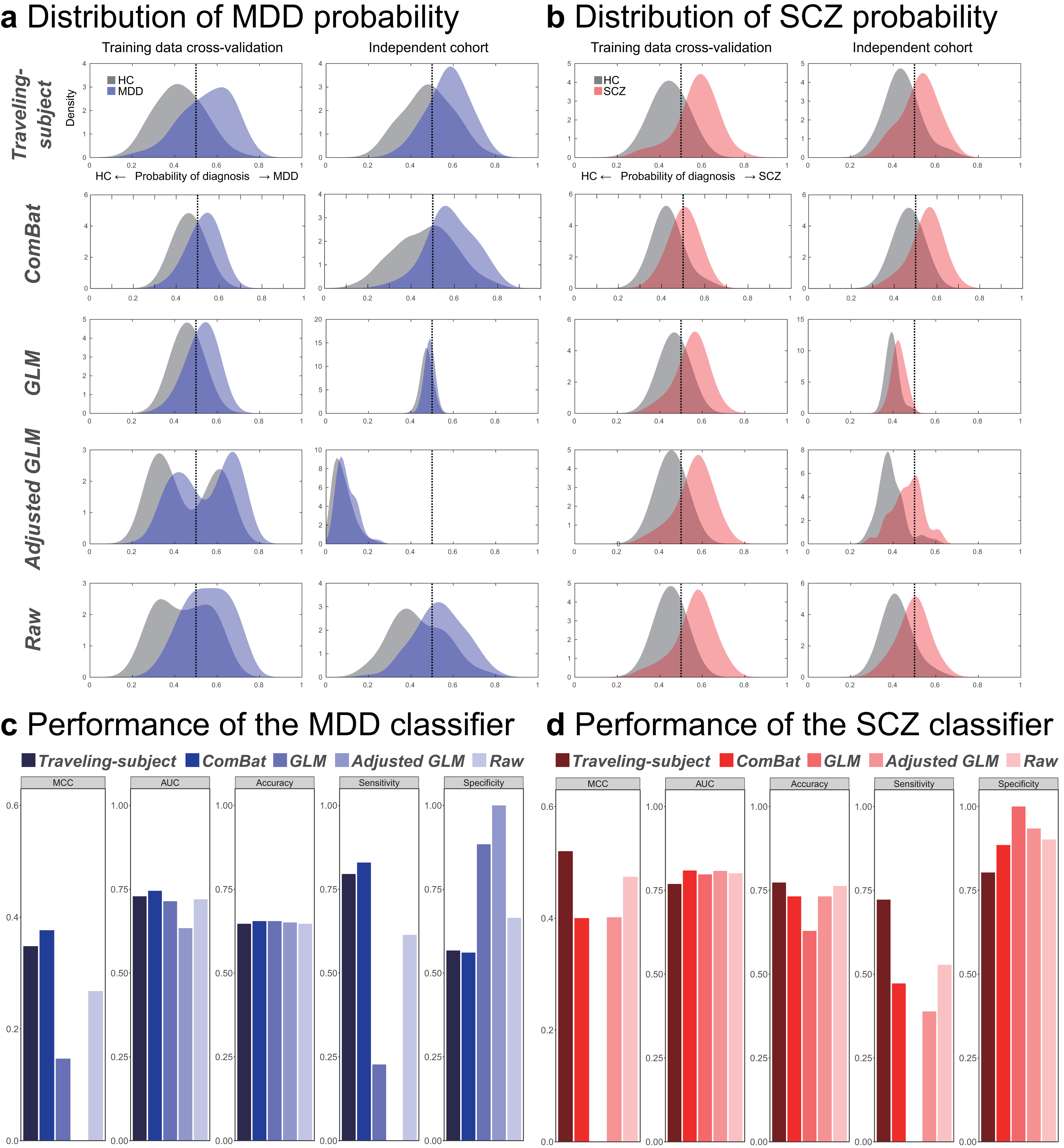

Supplement: S5 Fig — (A, B) Performance of each classifier in the training dataset for each harmonization method (blue for MDD, red for SCZ). Bars represent the average, whereas error bars represent the standard deviation across 100 resamplings. (C) Scatterplot of actual age and predicted age for each harmonization method. The solid line represents the linear regression of the actual age from the predicted age. The MAE and correlation coefficient (r) are also shown. Each data point represents one participant. AUC, area under the curve; MAE, mean absolute error; MCC, Matthews correlation coefficient; MDD, major depressive disorder; SCZ, schizophrenia. (TIF) [file pbio.3000042.s013.tif]

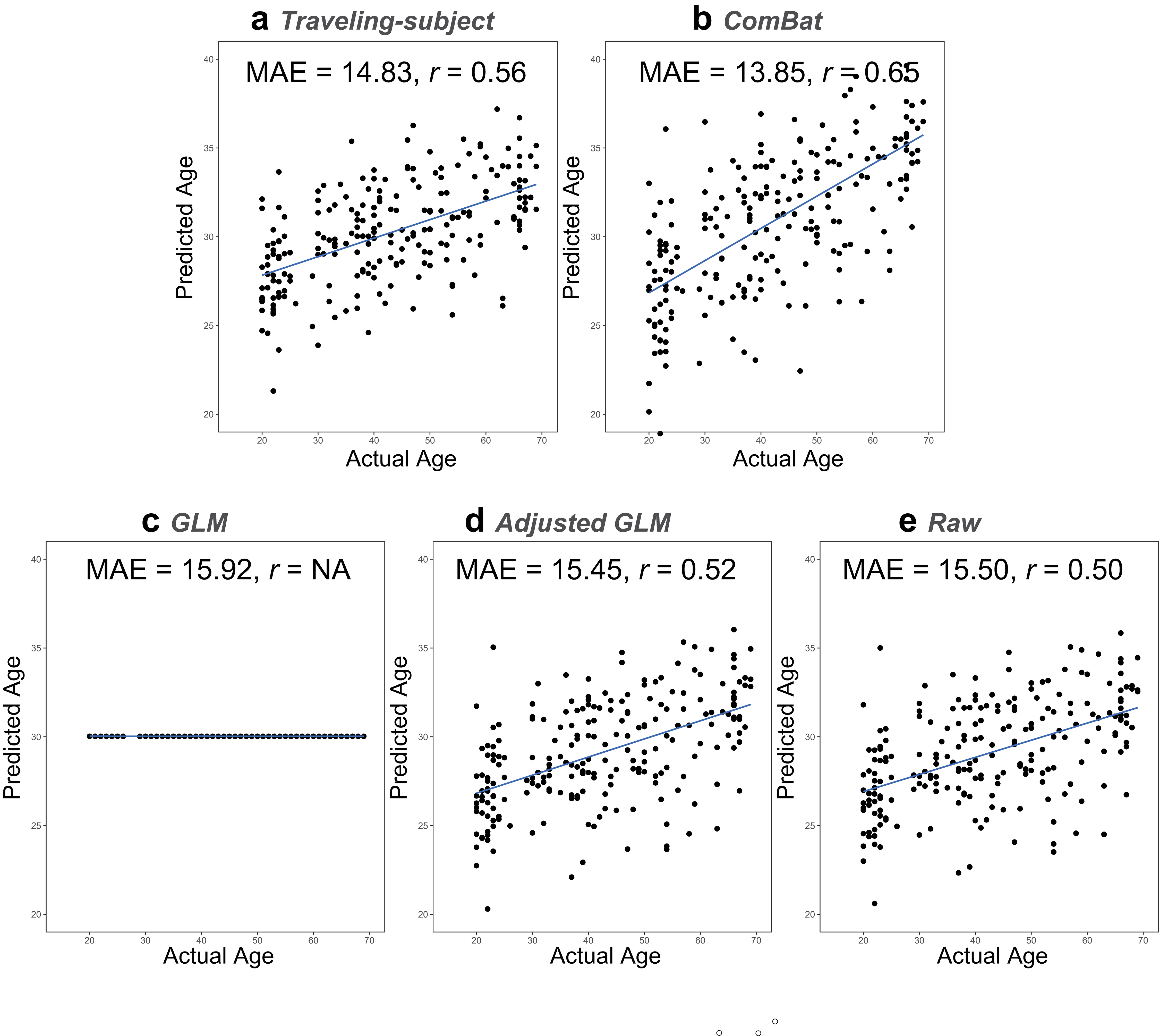

Supplement: S6 Fig — (A) The probability distribution for the diagnosis of MDD in the training dataset (left) and independent cohort (right) for each harmonization method. The MDD and HC distributions are depicted in blue and gray, respectively. (B) The probability distribution for the diagnosis of SCZ in the training dataset (left) and independent cohort (right) for each harmonization method. The SCZ and HC distributions are depicted in red and gray, respectively. (C, D) Classifier performance in the independent cohort for each harmonization method and each classifier (blue for MDD, red for SCZ). AUC, area under the curve; HC, healthy control; MCC, Matthews correlation coefficient; MDD, major depressive disorder; SCZ, schizophrenia. (TIF) [file pbio.3000042.s014.tif]

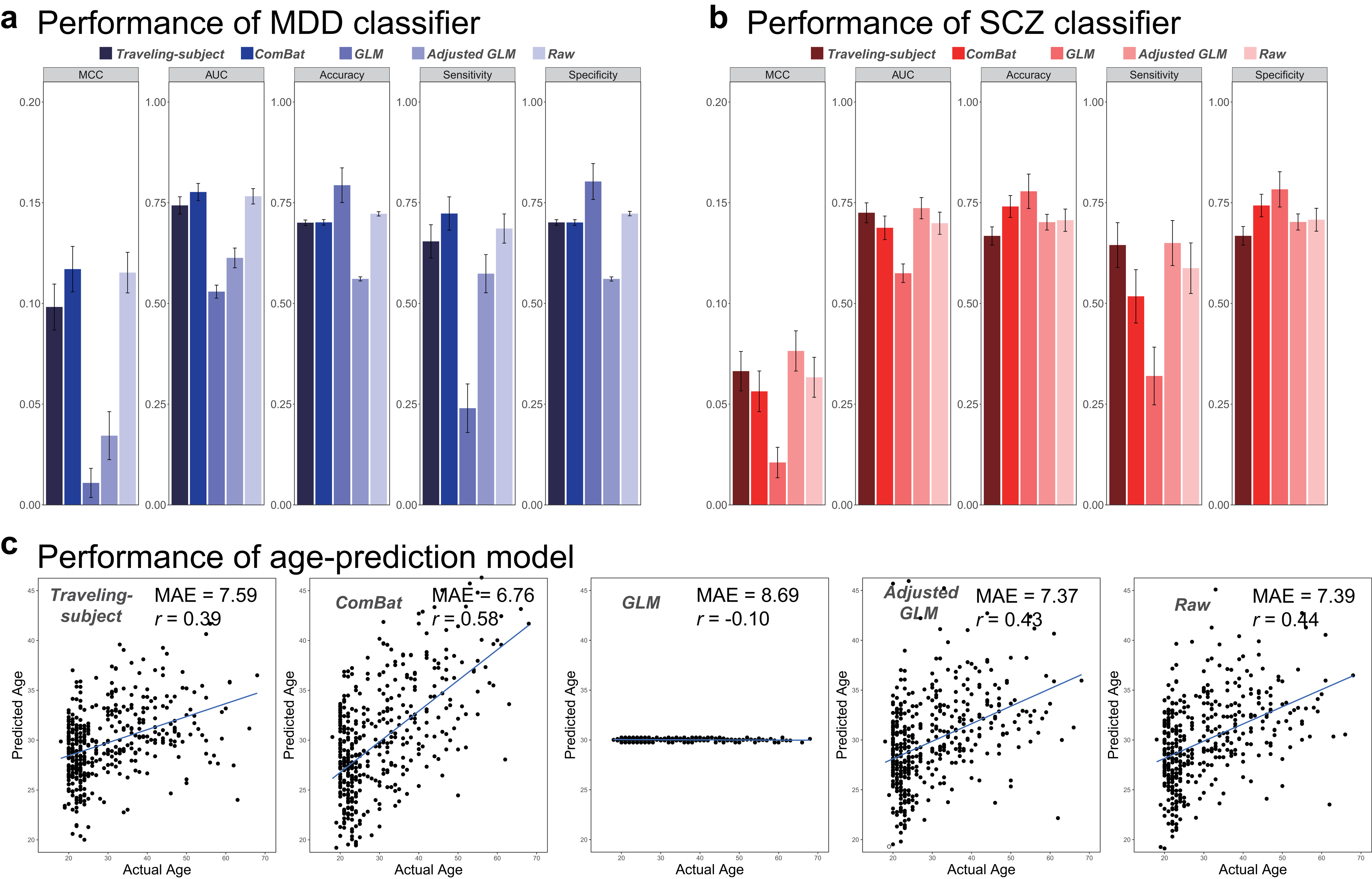

Supplement: S7 Fig — Scatterplots of actual age and predicted age. The solid line indicates the linear regression of the actual age from the predicted age. The MAE and correlation coefficient (r) are shown in each panel. Each data point represents one participant. Each panel shows the results for the (A) traveling-subject method, (B) ComBat method, (C) GLM method, (D) adjusted GLM method, or (E) raw method (i.e., the data were not harmonized across sites). GLM, general linear model; MAE, mean absolute error. (TIF) [file pbio.3000042.s015.tif]
